# Estimating the causal impact of non-pharmaceutical interventions on COVID-19 spread in seven EU countries via machine learning

**DOI:** 10.1038/s41598-025-88433-2

**Published:** 2025-03-17

**Authors:** Jannis Guski, Jonas Botz, Holger Fröhlich

**Affiliations:** 1 Department of Bioinformatics, Fraunhofer Institute for Algorithms and Scientific Computing (SCAI), Sankt Augustin, 53757 Germany; 2 University of Bonn, Bonn-Aachen International Center for Information Technology (b-it), Bonn, 53115 Germany

**Keywords:** Causal machine learning, Policy recommendation, Time-dependent confounding, Machine learning, Epidemiology

## Abstract

During the COVID-19 pandemic, Non-Pharmaceutical Interventions (NPIs) were imposed all over Europe with the intent to reduce infection spread. However, reports on the effectiveness of those measures across different European countries are inconclusive up to now. Moreover, attempts to predict the effect of NPIs in a prospective and dynamical manner with the aim to support decision makers in future global health emergencies are largely lacking. Here, we explore causal machine learning to isolate causal effects of NPIs in observational public health data from seven EU countries, taking into account specific challenges like their sequential nature, effect heterogeneity, time-dependent confounding and lack of robustness due to violated assumptions. In a pseudo-prospective scenario planning analysis, we investigate which recommendations our model would have made during the second wave of the pandemic in Germany, demonstrating its capacity to generalize to the near future and identifying effective NPIs. In retrospect, our approach indicates that a wide range of response measures curbed COVID-19 across countries, especially in the early phases of the pandemic. Interestingly, this includes controversial interventions like strict school and border closures, but also recommendation-based policies in Sweden. Finally, we discuss important data- and modeling-related considerations that may optimize causal effect estimation in future pandemics.

## Introduction

The global coronavirus disease 2019 (COVID-19) crisis has often been thought of as a large-scale natural experiment to study the effects that collective behaviour and public policy responses can have on the course that a respiratory pathogen pandemic takes (e.g., van de Weijer et al.^1^). So-called Non-Pharmaceutical Interventions (NPIs), such as confinement, school closures, travel regulations and mandatory mask wearing, are of special interest because their effect on disease spread has been discussed controversially and potential social and economic collateral damage has been a matter of heated debate.

However, the description as a natural experiment might be misleading because, for practical as well as ethical reasons, there can never be randomization as to when and where NPIs are imposed. In consequence, the effect that an NPI has on a pandemic situation is potentially subject to confounding bias. For example, virus spread may be accelerated in regions with high population density, while at the same time, decision makers may be counselled to give precedence to travel restriction policies for precisely these regions. Likewise, both pandemic spread and policy decisions may be influenced by time-dependent variables such as disease incidence, mobility in public spaces or policy-related sentiment in the population, which in turn may have been affected by earlier policies. This particular problem is referred to as time-dependent confounding^2^.

Mechanistic, statistical as well as machine learning techniques have been applied to retrospectively estimate the effectiveness of NPIs during the pandemic^3,4^. However, reports on the effectiveness of individual policies across Europe remain inconclusive up to now. In particular, the challenge to generalize findings even between different COVID-19 waves due to time-dependent confounding effects has been emphasized^5^. This has so far hindered the prediction of NPI effects in a prospective and dynamical manner with the aim to support decision makers in future global health emergencies.

In the following study, we suggest to leverage causal machine learning techniques to tackle confounding bias and isolate causal effects of NPIs on disease spread from observational public health data. Our contribution to the existing literature is threefold. First and foremost, we want to learn from data collected in seven European Union (EU) countries how causal machine learning can be best applied to the specific use case of NPI effects on disease spread. By the selection of a specific model architecture - adapted from the Counterfactual Recurrent Network (CRN) ^6^ -, we account for the particular challenges of effect heterogeneity over time and across regions, the sequential nature of observations and time-dependent confounding. We quantify model uncertainty and perform a comprehensive refutation analysis to monitor how robust our models are with respect to potential violations of standard assumptions. Pseudo-prospective scenario planning demonstrates which interventions our modeling approach would have recommended during an important period in the pandemic, namely the second wave in Germany. We investigate the models’ ability to generalize to the near future and identify driving features for effect prediction with SHapley Additive exPlanations (SHAP) analysis.

Secondly, we use the proposed model to estimate effects of different NPIs in retrospect, including strict interventions like stay-at-home orders and school closures across the seven countries, and also the much-discussed “Swedish strategy” of non-binding recommendations.

Finally, we derive from our analyses how modeling could be optimized and what data should be available to apply causal machine learning in order to understand policy effects if a comparable pandemic situation should come up in the future.

### Related work

#### Causal effect estimation with machine learning

A plethora of machine learning methods for causal effect estimation in the static setting has emerged in recent years^7–13^. In comparison, the setting of dynamic treatments and outcomes has been explored relatively little. Moraffah et al.^14 ^provide a review of existing methods that specifically target time-dependent confounding. In the recent past, a number of new approaches has been proposed^6,15–18^.

#### Models of NPI effectiveness

A comprehensive overview of approaches to NPI effectiveness estimation can be found in a paper by Banholzer et al.^19^. We focus here on studies that specifically applied causal machine learning.

Kristjanpoller et al.^20 ^estimated heterogeneous causal effects of policies in Chile with meta-learners^11 ^and causal forests^7^, and Barros et al.^21 ^applied covariate balancing to model mean population effects worldwide. However, both reinterpreted the data as static, thus neglecting challenges like sequential structure and time-dependent confounding. Kang and colleagues^22 ^addressed a related question, the causal effect of weather conditions on COVID incidence, with orthogonal forests^8^. Some studies tried to discover causal NPI-related relationships with methods like the SyPI algorithm^23 ^or structural equation models^24^.

To the best of our knowledge, we are the first to apply what Moraffah et al.^14 ^categorize as time-varying treatment effect estimation to answer the question of NPI effectiveness, thereby taking the problem of time-dependent confounding into account. This is particularly important as we - unlike many previous studies^5,25,26^ - explicitly adopt a vantage point *after* the pandemic and try to assess NPI effectiveness over the main pandemic years 2020–2022, for which a lot of variation can be expected in the confounders. We design our models such that bias due to changes in public mobility, vaccination rate and mask wearing habits is explicitly controlled over the whole observation period. Additionally, our implementation of refutation analysis provides a means to assess how robust estimates are both in retrospect and with regard to ongoing predictions in an upcoming wave.

## Materials and methods

### Causal framework

We build our study on the Neyman-Rubin potential outcomes framework^27^ and its adaptation to time-varying outcomes and treatments by Robins et al.^28^. Our goal is the prediction of $\tau$-step-ahead potential outcomes $Y_{t+\tau }(a_t)$, where $a_t$ is the treatment assignment (in our case whether an NPI is currently active or not) at time *t*. In the binary treatment case, causal treatment effects can be interpreted as the difference between the potential outcome $Y_{t+\tau }(1)$ (assuming treatment – i.e., NPI – is active at time *t*) and the potential outcome $Y_{t+\tau }(0)$ (assuming treatment is not active). In particular, we want to capture effect heterogeneity by conditioning the treatment effect on a history $H_t$, comprising past treatments, past and present outcomes, and past and present effect modifiers. Thus, what we want to estimate is the Conditional Average Treatment Effect (CATE) (equation (1)).

1 $$\begin{aligned} \mathbb {E}[Y_{t+\tau }(1) - Y_{t+\tau }(0) \mid H_t] \end{aligned}$$

It is the *fundamental problem of causal inference*^29^ that only the *factual* outcome $Y_{t+\tau } = a_t Y_{t+\tau }(1) + (1-a_t) Y_{t+\tau }(0)$ can be observed, but never the *counterfactual*. Nevertheless, counterfactual outcome distributions are identifiable if three standard conditions are fulfilled^17,28^:

1. **Sequential ignorability**: If conditioned on history $H_t$, the treatment is independent of the potential outcome: $A_t \perp \!\!\! \perp Y_{t+\tau }(a_t) \mid H_t$. This implies that there are no unobserved confounders beyond what is included in $H_t$.
2. **Sequential overlap**: The probability of receiving or not receiving a treatment must be non-zero for any observed realization of a history $h_t$ with $\mathbb {P}(H_t = h_t)> 0$: $0< \mathbb {P}(A_t = a_t \mid H_t = h_t) < 1$.
3. **Consistency**: If treatment $A_t = a_t$ is active, the factual (observed) outcome corresponds to the potential outcome under treatment $a_t$: $Y_{t+\tau }=Y_{t+\tau }(a_t)$.

In the data generation process for our study, sequential ignorability is given if we are able to include all features that have a substantial impact on both how fast COVID-19 spreads and whether a specific NPI is imposed at a given time and for a given region. For example, since we do not have access to surveys on the public compliance with NPIs across the board for any time and region, we have to assume that the confounding coming from this source is ignorable if other confounders like public mobility, vaccination policies etc. have already been included in the model.

The sequential overlap assumption is violated if there are no observations available from periods without active NPIs that are anywhere near comparable to periods with active NPIs. For instance, if school closures have only been active during the winter months with lower mean temperatures and lower relative humidity, causal models may make faulty predictions when extrapolating to the summer months in which mean temperatures and humidity are generally higher.

Consistency is given in our setting if the very same NPI does not have totally different qualities across different regions or at different times. If stay-at-home orders are rigorously enforced in one region, but not in another, and this leads to systematic differences in COVID spread between the regions, the assumption is violated.

### Data collection and pre-processing

#### Treatment: Non-pharmaceutical interventions

NPI data was retrieved from the Oxford COVID-19 Government Response Tracker (OxCGRT)^30^, where active policy responses are classified for any given day during the pandemic in pre-defined categories at country level worldwide. We focused on stay-at-home restrictions, school and workplace closures, as well as international and domestic travel regulations. Mask wearing policies and cancellations of public events or gatherings were neglected because in most countries, these were imposed once and then maintained throughout the pandemic (see Column A of Supplementary Fig. S2). In general, NPIs were binarized to no or non-mandatory policies in one and mandatory policies in the other treatment condition. For Sweden, which is known to have pursued a recommendation-based strategy to contain virus spread, stay-at-home regulations, internal travel regulations and mask wearing were considered “active” while mere recommendations were in place. Table 1 specifies the binarization criteria for each NPI.

**Table 1:** Binarized treatment levels of the NPI features from the OxCGRT.

NPI	Treatment level	
Required	0	1
Stay-at-home orders (npi_stay_home)	no measures or recommended only	required
School closures (npi_schools)	no measures or recommended only	required (at some or all levels)
Workplace closures (npi_work)	no measures, recommended only or required only by some	required for all but key workers
Internal movement restrictions (npi_internal_travel)	no measures or recommended only	movement restricted
Border closure (npi_international_travel)	no measures or screening, quarantine or bans on high-risk regions	total border closure

Recommended (Sweden only)	0	1
Stay-at-home recommendations (npi_stay_home_r)	no measures	recommended
Recommended internal movement restrictions (npi_internal_travel_r)	no measures	recommended
Recommended mask wearing (npi_masks_r)	no policy	recommended or required in some public spaces

#### Selection of countries and subnational units

Subnational regions in EU and partner countries are typically arranged along the Nomenclature of Territorial Units for Statistics (NUTS), which provides four levels of aggregation: NUTS 3 (small regions for specific analyses), NUTS 2 (basic regions for the application of regional policies), NUTS 1 (major socio-economic regions grouping together basic regions), and NUTS 0 (whole countries)^32^. Because NPIs were unavailable below country level, we only considered NUTS 0 and NUTS 1 in our analyses. It can be expected that interventions known for the national level will still have similar effects in the larger subnational aggregation units, but to a lesser extent when regions are subdivided further. Initially, we planned to include the ten largest EU countries by population, but had to discard Italy and Romania due to poor quality of the surveillance data, and the Czech Republic which is not subdivided below country resolution at NUTS level 1. This left us with seven EU countries (in order of population size): Germany with 16 NUTS 1 units (*Bundesländer*), France with eight units (*zones d’études et d’aménagement du territoire*, without overseas regions), Spain with seven units (*agrupaciones de comunidades autónomas*), Poland with six units (*regiony*), the Netherlands with four units (*landsdelen*), Belgium with three units (*gewesten* or *régions*), and Sweden with three units (*grupper av riksområden*). See Supplementary Fig. S1 for a summary of the time periods for which data was available per country, and Column A in Supplementary Fig. S2 for an overview of the periods during which specific NPIs were active in each country.

#### Outcome: Time-varying effective reproduction number

As an indicator of virus spread at a given time, we derived the time-varying effective reproduction number $R_t$ from public surveillance data. $R_t$is a measure that quantifies the average number of secondary infections caused by a single infected individual at a specific point in time during a pandemic^33^. If $R_t$ is greater than 1, it indicates that the epidemic is growing, while values less than 1 suggest a decline in the spread of the disease.

We estimated $R_t$for all countries based on daily numbers of confirmed cases provided at NUTS level 3 by the COVID-19 European regional tracker^34^, which we aggregated to NUTS levels 0 and 1, respectively. $R_t$ estimates were then obtained with an implementation of a method by Cori et al.^33^ in the epyestim^35^ Python package, using the covid19 preset and smoothing windows of 28 days. To account for the lag between infection and case confirmation, the covid19 preset assumes a delay distribution as reported by Brauner et al.^26^ (see Supplementary Fig. S3).

As we expect that $R_t$ is reduced in NPI active conditions, CATE estimates below zero hint at effective policies.

Since the recording of case numbers is subject to variations over time and across regions due to changes in testing capacities and policies, we alternatively estimated $R_t^{deaths}$on the basis of COVID-related death numbers. We retrieved this data from national platforms for all seven countries, and modelled the lag between infection and death with a log-normal distribution as proposed by Ward and Johnsen^36^ (see Supplementary Fig. S3).

#### Confounding and effect-modifying variables

Table 2 summarizes all additional variables that were considered as confounders or effect modifiers, their temporal and spatial resolutions, units, and data sources. Additional details on the features and their preprocessing can be found in Supplementary Methods S1.

**Table 2:** Overview of confounding / effect-modifying variables. ECDC, European Centre for Disease Prevention and Control; IHME, Institute for Health Metrics and Evaluation; ECA&D, European Climate Assessment & Dataset; IPTCC, PREDICT index of climatic transmissibility of COVID-19; EEA, European Environment Agency.

	Feature	Source	Unit	Temporal resolution	Spatial resolution
COVID-related	Tests	ECDC	per 100,000	Weekly	NUTS 0
	Mask use projection	IHME^*^	%	Daily	NUTS 0/1
	Vaccination policy	OxCGRT	categories	Daily	NUTS 0
Mobility	Google mobility trends	Google	% deviation from ref.	Daily	NUTS 1
	Commuters (foreign)	Eurostat	per 100,000	Annual	NUTS 1/2
	Commuters (outregion)	Eurostat	per 100,000	Annual	NUTS 1/2
Meteorological	Mean temperature	ECA&D		Daily	Arbitrary
	Relative humidity	ECA&D	%	Daily	Arbitrary
	Precipitation amount	ECA&D	0.1 mm	Daily	Arbitrary
	IPTCC^37^		%	Daily	Arbitrary
Air pollution	Nitrogen Dioxide (NO2)	EEA	$\upmu$g/m3	Daily	Arbitrary
	Ozone (O3)	EEA	$\upmu$g/m3	Daily	Arbitrary
	Particulate Matter (PM10)	EEA	$\upmu$g/m3	Daily	Arbitrary
Socioeconomic	Gross domestic product	Eurostat	EUR per capita	Annual	NUTS 3
	Hospital beds	Eurostat	per 100,000	Annual	NUTS 2
	Physicians	Eurostat	per 100,000	Annual	NUTS 2
	Broadband internet	Eurostat	% of households	Annual	NUTS 1
Demographic	Population density	Eurostat	inhabitants/km2	Annual	NUTS 3
	Population median age	Eurostat	years	Annual	NUTS 3

^*^Incorporated data from the Delphi Group at Carnegie Mellon University and University of Maryland COVID-19 Trends and Impact Surveys (in partnership with Facebook), the Kaiser Family Foundation and the YouGov COVID-19 Behaviour Tracker survey.

### Study design

#### Model architectures

**Fig. 1:**
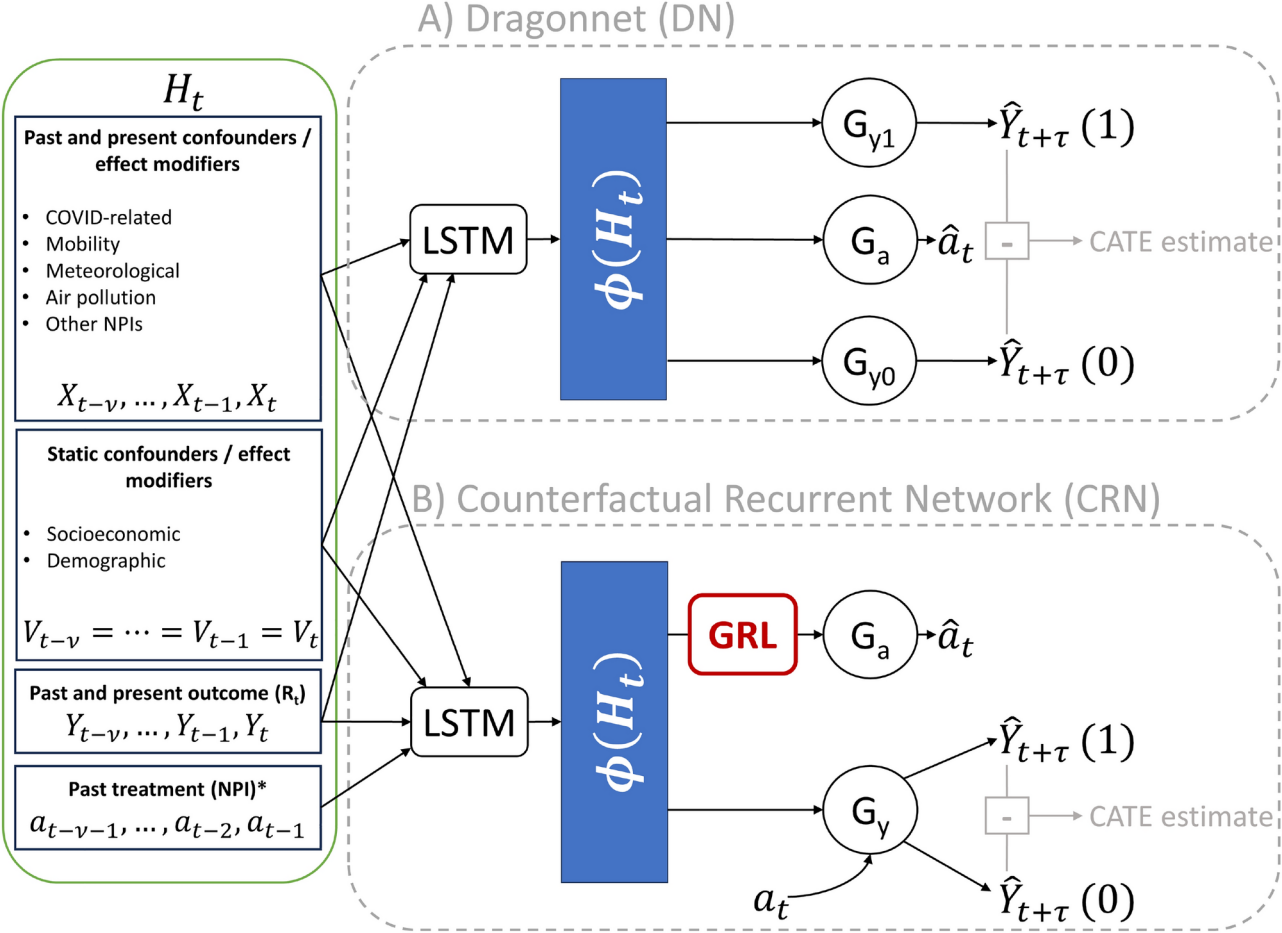
Overview of model architectures, inputs and outputs. Left: All inputs for the neural network models are summarized in $H_t$. Top: Model architecture of the Dragonnet (DN). Bottom: Model architecture of the Counterfactual Recurrent Network (CRN). * Past treatment is used as input only by the CRN, not by the DN.

Our modeling is based on recurrent neural networks to account for the sequential nature of the data. Specifically, we compare two model architectures for CATE prediction (see Fig. 1) to demonstrate the importance of addressing time-dependent confounding. Both map the history $H_t$ at a given time-point *t* and region into a space of latent representations $\Phi (H_t)$, from which the potential outcomes $Y_{t+\tau }(1)$ and $Y_{t+\tau }(0)$ - namely $R_t$ or $R_t^{deaths}$ - at time point $t+\tau$ are predicted. For sequence lengths $\nu$, $H_t$ includes past and present time-dependent confounders $X_{t-\nu },...X_{t-1},X_{t}$ such as COVID-19-related, mobility-related, meteorological and air pollution features as well as NPIs not considered as treatment, past and present outcomes $Y_{t-\nu },...Y_{t-1},Y_{t}$, past treatments $a_{t-\nu -1},...a_{t-2},a_{t-1}$ and static confounders $V_{t-\nu }=...=V_{t-1}=V_{t}$, i.e., socioeconomic and demographic features that do not vary over the modelled time span. The two architectures differ significantly in the way the latent representations $\Phi (H_t)$ are learned.

The first architecture (illustrated in the top half of Fig. 1) is a version of the Dragonnet (DN)^13^ with a Long Short-Term Memory (LSTM) module to encode the input sequences. Our DN has two heads $G_{y1}$ and $G_{y0}$ to predict the potential outcomes $Y_{t+\tau }(1)$ and $Y_{t+\tau }(0)$ ; in $G_{y1}$, during training, weights are updated only for examples from the treatment active condition, and only for examples from the control condition in $G_{y0}$. CATE estimates are obtained by passing an input sequence through the network and subtracting the output of $G_{y0}$ from the output of $G_{y1}$. A third head $G_a$ with a sigmoid activation function is trained to predict the current treatment assignment $a_t$ (NPI of interest at time *t*). Thereby, parts of the input that are not relevant for the prediction of treatment assignment are discarded in the learned latent representation, following the argument that those parts are also not relevant for the prediction of the causal effect^13^. As a model developed for the setting of static observations, the DN does not have a mechanism to deal with time-dependent confounding.

The second architecture (illustrated in the bottom half of Fig. 1) is an adaptation of the Counterfactual Recurrent Network (CRN) proposed by Bica et al.^6^, more specifically the encoder part. Like the DN, the CRN also encodes input sequences from $H_t$ with an LSTM block to a latent representation $\Phi (H_t)$, which is then passed on to predict the outcome, and has a head $G_a$ with sigmoid activation that is trained to predict the current treatment assignment $a_t$. However, a Gradient Reversal Layer (GRL) between treatment head and representation layer ensures that $\Phi (H_t)$ is not predictive of $a_t$, while retaining the ability to predict the outcome. Thus, the learned representation is supposed to be treatment-invariant and to uncouple the link between the history and current treatment assignment for a given observation, thereby reducing the bias arising from time-dependent confounding. Sticking to the architecture as described in the original publication, our CRN has only one head $G_y$ for potential outcomes prediction which is trained with all example sequences, regardless of their treatment condition. To get CATE estimates, $a_t$ is included as an input feature and set to one or zero to estimate the two potential outcomes.

We used mean-squared error as partial loss for all outcome prediction heads, and binary cross-entropy for the treatment heads. A parameter $\lambda$ governed the trade-off between these two partial losses in the overall loss; $\lambda$ is consistently at one for DN models, and increased with an exponential schedule for CRN as described by Bica et al.^6^. The GRL in the CRN performs the simple trick of multiplying its inputs by −1 during backpropagation, thereby leading the model to perform gradient ascent instead of descent on the cross-entropy loss of $G_a$ with respect to the weights for $\Phi (H_t)$, while maintaining gradient descent for the weights in $G_a$ itself^38^.

Each model was fitted for only one NPI as treatment at a time, with the other NPIs included as additional confounders (and, for the CRN, the history of the NPI of interest). While the CRN can theoretically be fitted for multiple treatments, this is not promising for our use case where NPIs are often imposed in groups at a time and can thus be expected to be confounded not only in the history sequences, but also at present.

We trained models country-wise and systematically compared models for 7- vs. 14-days ahead predictions ($\tau =7$ vs. $\tau =14$), and NUTS level 0 units only vs. combined level 0 plus 1 units with respect to CATE predictions, factual outcome prediction in an evaluation period, and robustness under refutation testing.

#### Hyperparameter search and model fitting

For every country, the last three months of the available observation period from the whole pandemic were withheld for evaluation and the month before that for validation (see Supplementary Fig. S1). Only if outcome prediction performance is reasonable also on unseen future data one can be confident that the models do not overfit on random noise from the training data or, even worse, from the initialization of the latent representations. Everything before these four months was used for hyperparameter search and refitting once the best hyperparameters had been identified.

We optimized hyperparameters with Optuna^39^ and following a time-series cross validation scheme. Training and validation periods moved forward through time, with the training set spanning at least 150 days, and the validation set the 28 days after the training set (see Supplementary Fig. S1). The tree-structured Parzen estimator was selected for sampling from the parameter space (see Supplementary Table S2), and performance with one set of parameters evaluated based on a weighted average of validation losses at the best epochs over the cross validation folds. Later folds got linearly increasing weights because they can be expected to be more similar to the complete data. Eventually, models to explore NPI effectiveness in retrospect across the complete available observation periods from the pandemic years were fitted on the whole training data with the final set of hyperparameters, using the withheld one month for validation. Likewise, models for retrospective analysis were trained only on the observation period of the first wave of infections in 2020 and the summer months afterwards, i.e., calendar weeks 11 to 35. In this case, the validation set spanned weeks 36 to 40.

Both during hyperparameter search and the final fitting, early stopping was applied based on the respective validation periods. If validation loss had stalled or decreased for 50 epochs in a row, training was stopped and the model weights with the best validation loss restored. Models were trained for 250 epochs at the most.

To increase the number of sequences, we made use of a sliding window approach. All time series were subdivided into pairs of fitting windows and directly adjoining prediction windows, with offsets of one day between pairs. The length $\nu$ of the fitting windows was treated as a hyperparameter (and optimized between 7, 14 and 21 days), the length of the prediction windows $\tau$ was 7 or 14 depending on whether the CATE estimates were formed based on one- or two-week ahead predictions.

Prior to entering the model, inputs were standardized using mean and standard deviation statistics per feature from the training data.

Confidence intervals of model predictions were obtained via the variational dropout method^40^, a dropout objective to approximate the posterior of a deep Gaussian process as is usual in Bayesian inference.

#### Refutation analysis

The evaluation of causal machine learning models differs significantly from that of conventional predictive ones. More specifically, for a causal model, it is not the primary objective to reach a high predictive performance on unseen test data, but to obtain reliable estimates of causal effects. Since there is no way of knowing the true CATE values in real-world data, it is generally recommended to check the robustness of causal models under different data manipulations that refute crucial assumptions^41^. We implemented versions of common refutation tests from Sharma et al.^41^ specifically for sequential data and for CATE estimates. Random realizations of each manipulation are applied to the data in 100 simulations, and the CATE point estimates for each simulation make up test distributions for every time point in each region.

1. **Data Subset Refutation**^41^ tests if the dataset is too small for robust effects, i.e., it checks the variance of the causal estimates. The manipulation is to sample 80 % of fitting/prediction window pairs without replacement for training the model. To pass the test, the original point estimate of the CATE should not be outside of the test distribution and if the original CATE interval estimate lies below zero, the test distribution should not cover zero either.
2. **Random Common Cause Refutation**^41^ tests the assumption of (sequential) ignorability / no unobserved confounding. A random walk variable – generated as the cumulative sum of steps drawn from a standard normal distribution – is added to the data for each region. To pass the test, the original point estimate should not be outside of the test distribution and if the original CATE interval estimate lies below zero, the test distribution should not cover zero either. Note that this is still no guarantee that unobserved confounding is not a problem. The best way to ensure ignorability is to consult expert knowledge about which features are essential to include as confounders; if some of these are disregarded, bias may persist that is undetected by random common cause refutation. However, the other way around, failing the test can reveal that the ignorability assumption is clearly violated and indicate that caution is required when interpreting the causal effect estimates.
3. **Placebo Treatment Refutation**^41^ tests if the observed effect is not specific to treatment assignment and would be predicted for most chronologies of treatments. We shift the observed treatment variable circularly (elements that are shifted out from the end of the available period are added back to the beginning) for each region. For a given observation to pass the test, zero should not be above the test distribution.
4. **Propensity Thresholding**^43^ tests the assumption of (sequential) overlap and treatment class imbalance. It is not a refutation test in the narrower sense because it does not depend on repeated simulations. Treatment assignment is predicted by the fitted model; the output probabilities should not be below 0.1 or above 0.9. Otherwise, the overlap assumption may be violated (making it too easy for the model to predict treatment assignment), or the treatment condition groups too imbalanced (making it attractive to exclusively predict the more frequent treatment condition as a strategy for loss minimization).

#### Pseudo-prospective scenario planning

Finally, we simulated which predictions our models would have made regarding NPI effectiveness at a specific critical point in the pandemic. For this, we considered the second wave in Germany, retrospectively dated between week 40/2020 and week 8/2021^44^. At that time, most NPIs had already been active during the first wave, and the once more increasing numbers of confirmed cases demanded a new NPI strategy while vaccines were not yet available. The second wave was used as evaluation period, and all available data before that as training period. Again, the final month of the training period served as validation set for early stopping. We excluded npi_work and vaccination policy for these analyses, since both remained constantly at level 0 throughout the training period. By evaluating factual outcome prediction in the evaluation period, we checked if the models were capable of generalizing predictions to the near future. CATE was estimated and refuted for both training and evaluation period. Eventually, SHAP analysis was performed to find out which features were particularly influential for CATE predictions during the second wave. In spite of criticism that SHAP values do not always reliably attribute the correct importance to features^45–48^, they are still a common and simple way to understand which features play an important role in a model’s predictions. By using the KernelExplainer from the shap^49^Python package, we factored in Janzing et al.’s^47^ argument that *unconditional* rather than *conditional* distributions should be used in the process of feature dropping.

Germany was chosen for a proof of concept because this work has been funded by the German government with the aim of developing a system for early warning, monitoring and decision support specifically in Germany. Additionally, with 16 subnational units, Germany is the country with the largest amount of training data when taking NUTS level 1 into account, and surveillance data seemed to have a comparably good quality in pre-study inspection. Moreover, we restricted the pseudo-prospective scenario planning to CATE based on $R_t$ (and not $R_t^{deaths}$) because it used to be one of the main metrics monitored by the state authorities during the pandemic years.

## Results

### Model robustness

**Fig. 2:**
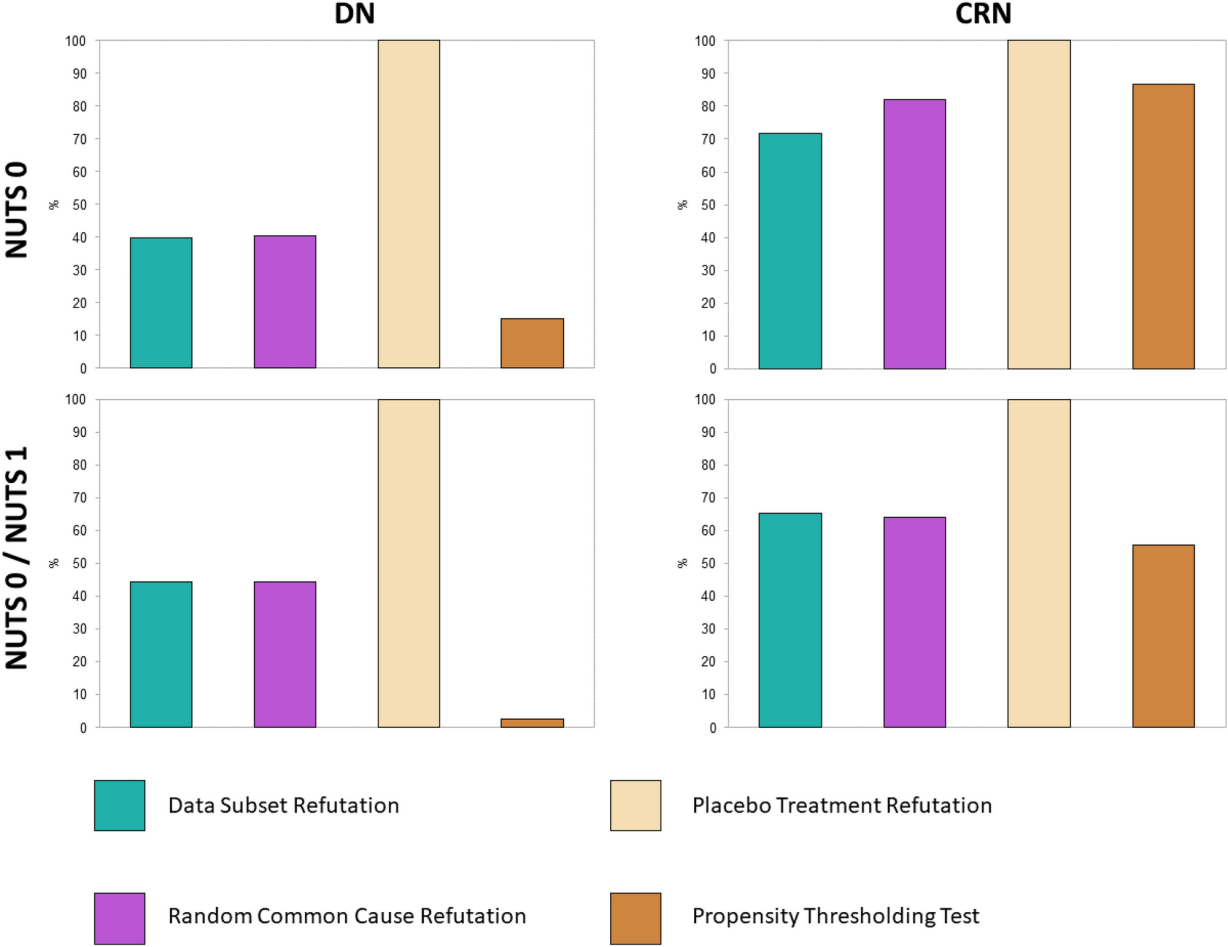
Model robustness according to refutation analysis. Portions of observations that passed each refutation test in data withheld for evaluation, comparing DN (left) vs. CRN (right) and NUTS level 0 only (top) vs. levels 0 and 1 combined (bottom).

Fig. 2 summarizes the percentages of observations from evaluation data that passed each of the refutation tests, aggregated over the retrospective $R_t$ models for all countries and NPIs. An analogous figure for the training data is given in Supplementary Fig. S4.

CRN models seem generally more robust than DN models, especially with respect to propensity thresholding. A closer look at the treatment assignment predictions reveals that for the CRN, propensity thresholds were mostly exceeded due to the model learning to emit probabilities close to zero or one regardless of true treatment assignment; this might have been a strategy to minimize loss in the face of high imbalance between NPI active and non-active conditions. For the DN, on the other hand, treatment assignment was perfectly predicted most of the time, indicating severe overlap violation that is carried from the original into the latent feature space. However, high correlations between propensity thresholding test and both data subset and random common cause refutation (0.25 / 0.24 for training, 0.17 / 0.17 for evaluation period in DN, 0.62 / 0.63 for training, 0.58 / 0.59 for evaluation period in CRN) reveal that failing this test is generally detrimental for model robustness, regardless of its cause. As Fig. 2 refers to evaluation data, we can rule out that propensity thresholds were systematically surpassed due to overfitting.

Training models only with NUTS level 0 data produced more robust CATE estimates than combining NUTS 0 and 1. The reason for this may be an additional exacerbation of class imbalance, or poor quality data at subnational level in some countries.

Unspecific treatment effect estimates regardless of the NPI chronology did not seem to be a problem, as almost all observations passed the placebo treatment refutation test for all models.

Since the treatment-invariant representation in the CRN was found to effectively mitigate violations of the overlap assumption and produce more robust predictions altogether, we will focus on results obtained with this architecture in the following.

### Retrospective CATE estimation

Supplementary Table S3 shows the retrospective models’ performances for $R_t$ and $R_t^{deaths}$ prediction in the training and evaluation periods for the whole available observation periods, aggregated over countries and NPIs. The models did not overfit significantly. At NUTS 0+1 level, the prediction of $R_t$ was notably more accurate compared to $R_t^{deaths}$. Due to the larger gaps between training and evaluation performance for 14-days ahead prediction for both $R_t$ and $R_t^{deaths}$, we will narrow our report down to estimates seven days into the future.

**Fig. 3:**
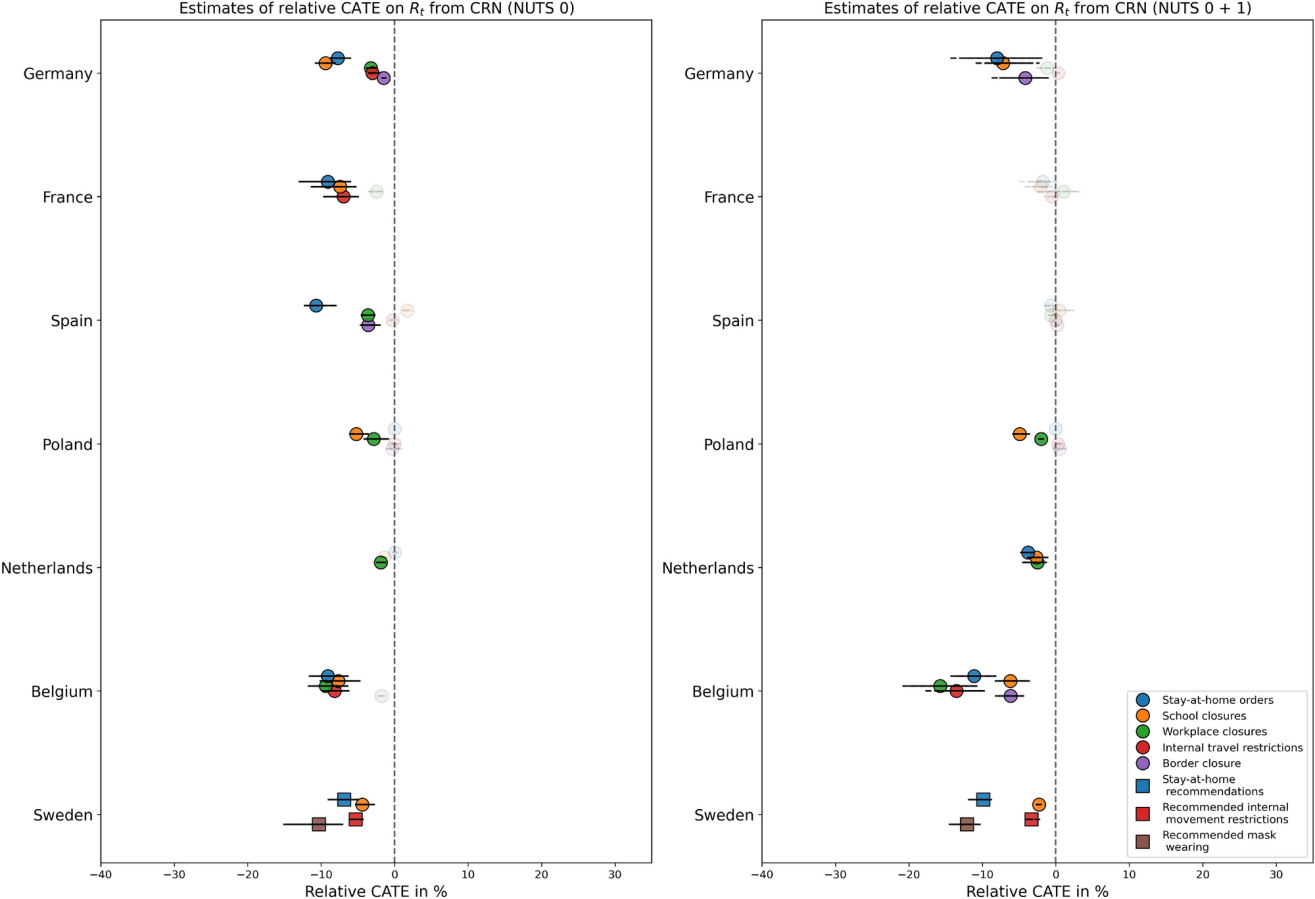
Distributions of predicted relative CATE on $R_t$ from CRN over time per NPI and per country. Left: NUTS level 0 only, right: Fitted with levels 0 and 1 combined, but predictions only for NUTS 0. Solid lines denote the 95 % CI of point estimates over time, dashed lines the 95 % CI of lower and upper boundaries of interval estimates over time. Markers are greyed out if CATE is non-significant or any refutation test is failed by 50 % or more observations.

Fig. 3 provides a high-level overview of distributions for predicted relative CATE on $R_t$ per NPI and country over the complete training periods from the pandemic years 2020 to 2022. Relative CATE on $R_t$ is reported to make sure that effect estimates are not driven by varying absolute $R_t$ over time and across countries, and is defined as the predicted CATE divided by the predicted potential outcome $Y_{t+\tau }(0)$, both with respect to $R_t$. It was found to be distributed below zero for almost all NPIs, i.e., NPIs were estimated to be effective, although effect sizes were only moderate (mostly above −15 %). This includes both strict mandatory policies across countries and recommendation-based measures in Sweden.

Estimated distributions of relative CATE on $R_t$ are always rather narrow, which could either indicate a relatively constant effect over the whole pandemic or limitations of our models to learn the heterogeneity of NPI effects over time. When including NUTS level 1 next to level 0, more heterogeneous effects were predicted for Germany, which has the highest number of NUTS 1 units among all countries. This may indicate that subnational data can help to model the variation of NPI effectiveness over time. On the other hand, some effects observed at NUTS level 0 vanished when level 1 data was included.

**Fig. 4:**
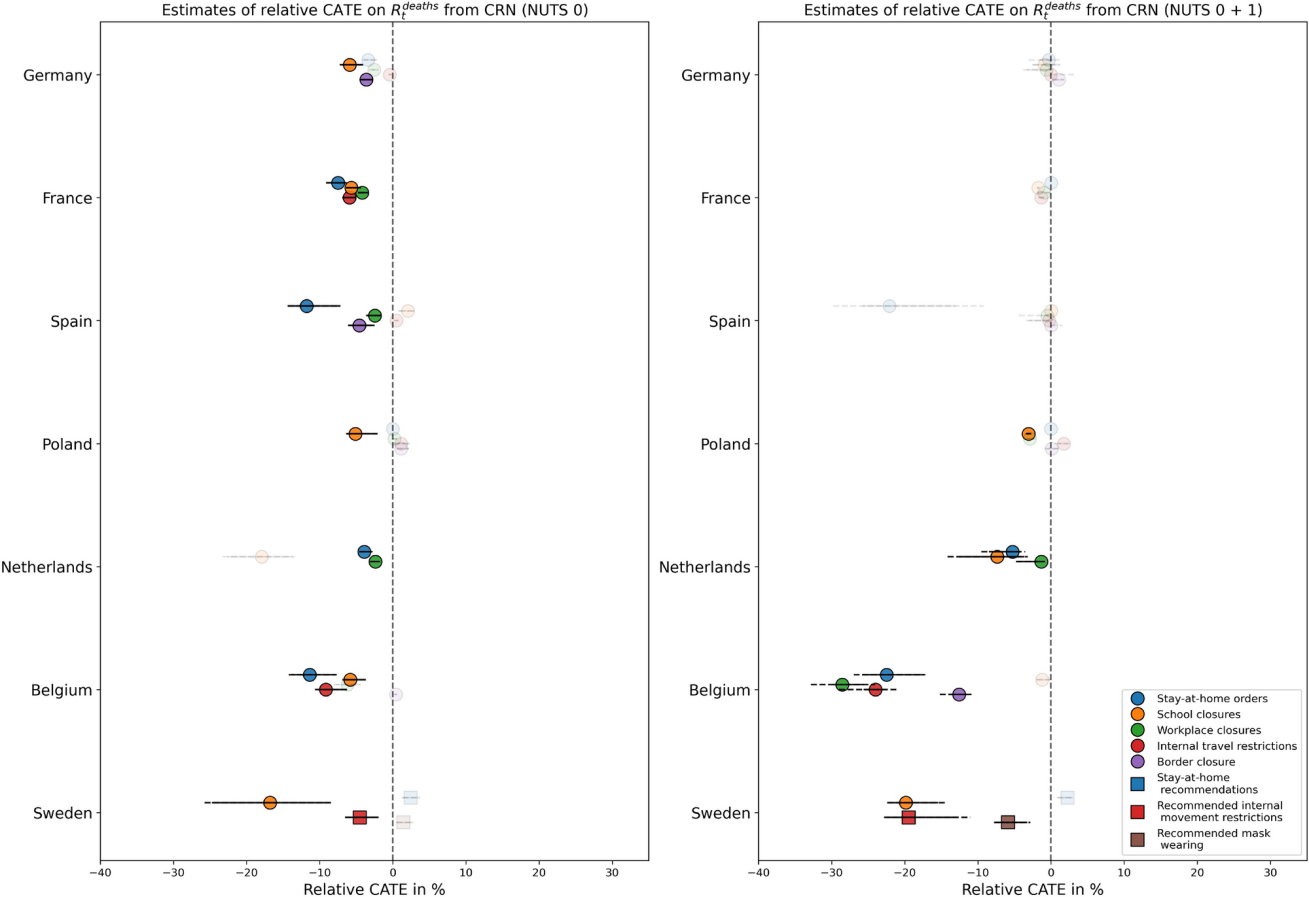
Distributions of predicted relative CATE on$R_t^\textbf{deaths}$ from CRN over time per NPI and per country. Left: NUTS level 0 only, right: Fitted with levels 0 and 1 combined, but predictions only for NUTS 0. Solid lines denote the 95 % CI of point estimates over time, dashed lines the 95 % CI of lower and upper boundaries of interval estimates over time. Markers are greyed out if CATE is non-significant or any refutation test is failed by 50 % or more observations.

As shown in Fig. 4, results are generally similar for predicted relative CATE on $R_t^ {deaths}$. However, some effects observed for $R_t$ cannot be replicated (e.g., all NPIs in Germany and stay-at-home recommendations in Sweden), while others become more pronounced (e.g., all NPIs except school closures in Belgium, school closures in Sweden, with effect sizes of −20 to −30 %).

**Fig. 5:**
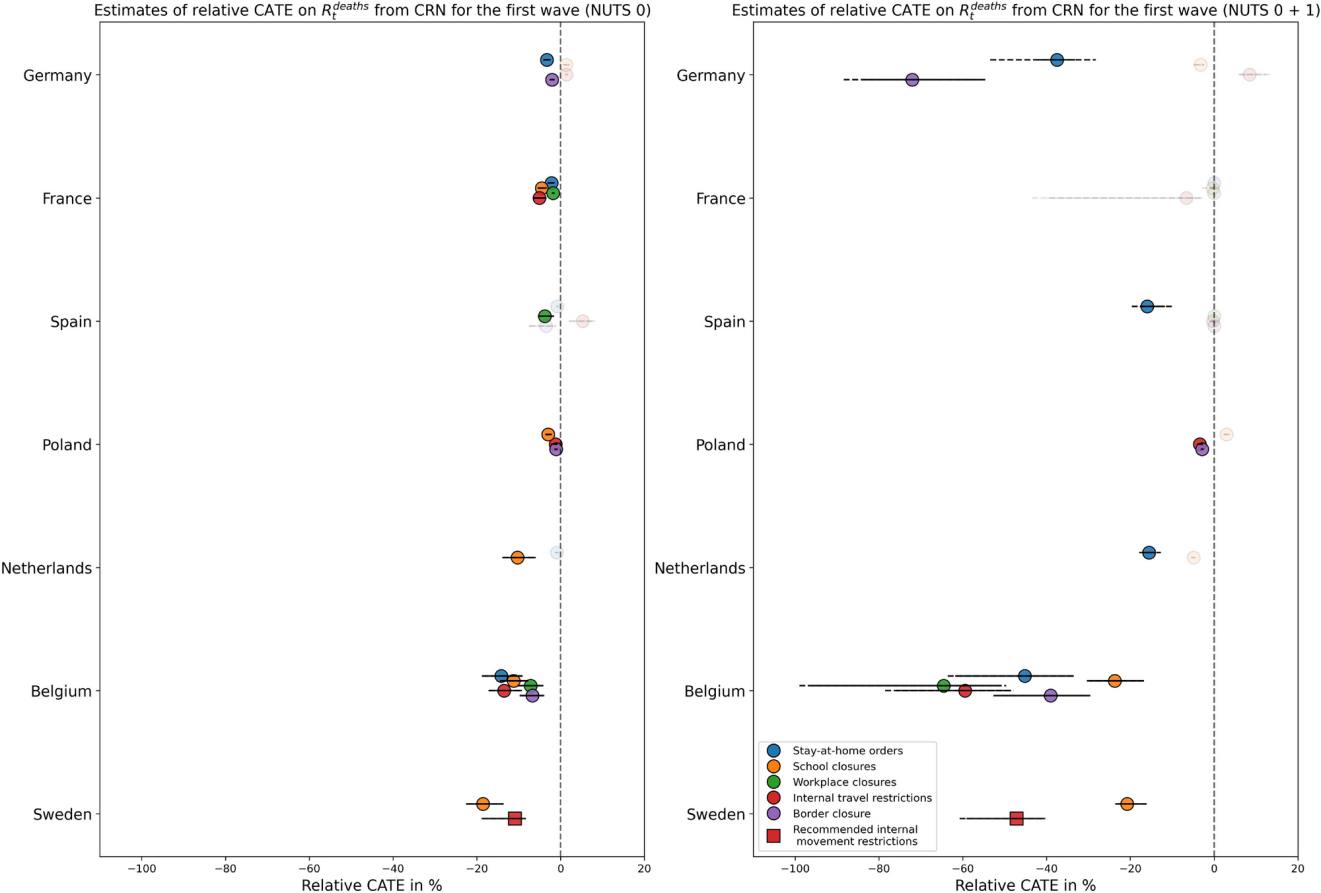
Distributions of predicted relative CATE on $R_t^{\textbf{deaths}}$ in the first wave from CRN over time per NPI and per country. Left: NUTS level 0 only, right: Fitted with levels 0 and 1 combined, but predictions only for NUTS 0. Solid lines denote the 95 % CI of point estimates over time, dashed lines the 95 % CI of lower and upper boundaries of interval estimates over time. Markers are greyed out if CATE is non-significant or any refutation test is failed by 50 % or more observations.

Effect estimates for some NPIs are decisively larger when narrowing down the analysis to the first wave of the pandemic, as can be seen in Fig. 5 for $R_t^{deaths}$. Note that the x-axis has been scaled down compared to Fig. 3 and 4, and that only NPIs are included that have actually been active at some point during the first wave (see Supplementary Fig. S2). A larger number of effects lie below −20 % for models fitted on combined NUTS level 0 and 1 data, with some policies (border closure in Germany, workplace closures and internal travel regulations in Belgium) having a predicted effect consistently below −50 % during most of the observation period. To a lesser extent, effect sizes are also increased in the first wave for $R_t$ compared to Fig. 3, which is shown in Supplementary Fig. S5.

### Pseudo-prospective scenario planning

In our pseudo-prospective scenario planning for the second wave in Germany (optimized hyperparameters can be found in Supplementary Table S2), Table 3 demonstrates that the models were able to generalize $R_t$ predictions to the unseen evaluation data and to provide robust CATE estimates under refutation. All NPIs are recommended as policies for the second wave with the exception of internal movement restrictions, for which CATE estimates were only marginally (albeit robustly) below zero and the pass rate of 0 % in the propensity thresholding test hints at problems with too few observations without active NPI.

**Table 3:** CATE estimates, factual outcome prediction performance and refutation results per NPI in pseudo-prospective scenario planning. CI, Confidence Interval; SD, Standard Deviation; MAPE, Mean Absolute Percentage Error (see definition in Supplementary Table S3); DSR, Data Subset Refutation; RCCR, Random Common Cause Refutation; PTR, Placebo Treatment Refutation; PTT, Propensity Thresholding Test.

NPI	CATE (second wave) (MEAN (95% CI))	Outcome prediction (MEAN (SD))		Refutation results (second wave)			
		MAPE (training)	MAPE (second wave)	DSR	RCCR	PTR	PTT
npi_stay_home	−0.13 (−0.11,−0.16)	9.62% (± 0.1%)	13.05% (± 0.1%)	91.4%	98.7%	100.0%	100.0%
npi_schools	−0.19 (−0.17,−0.20)	9.63% (± 0.1%)	13.04% (± 0.1%)	100.0%	100.0%	100.0%	100.0%
npi_internal_travel	−0.03 (−0.03,−0.04)	11.95% (± 0.1%)	8.34% (± 0.1%)	100.0%	100.0%	100.0%	0.0%
npi_international_travel	−0.18 (−0.16,−0.19)	9.26% (± 0.1%)	8.60% (± 0.1%)	100.0%	100.0%	100.0%	100.0%

**Fig. 6:**
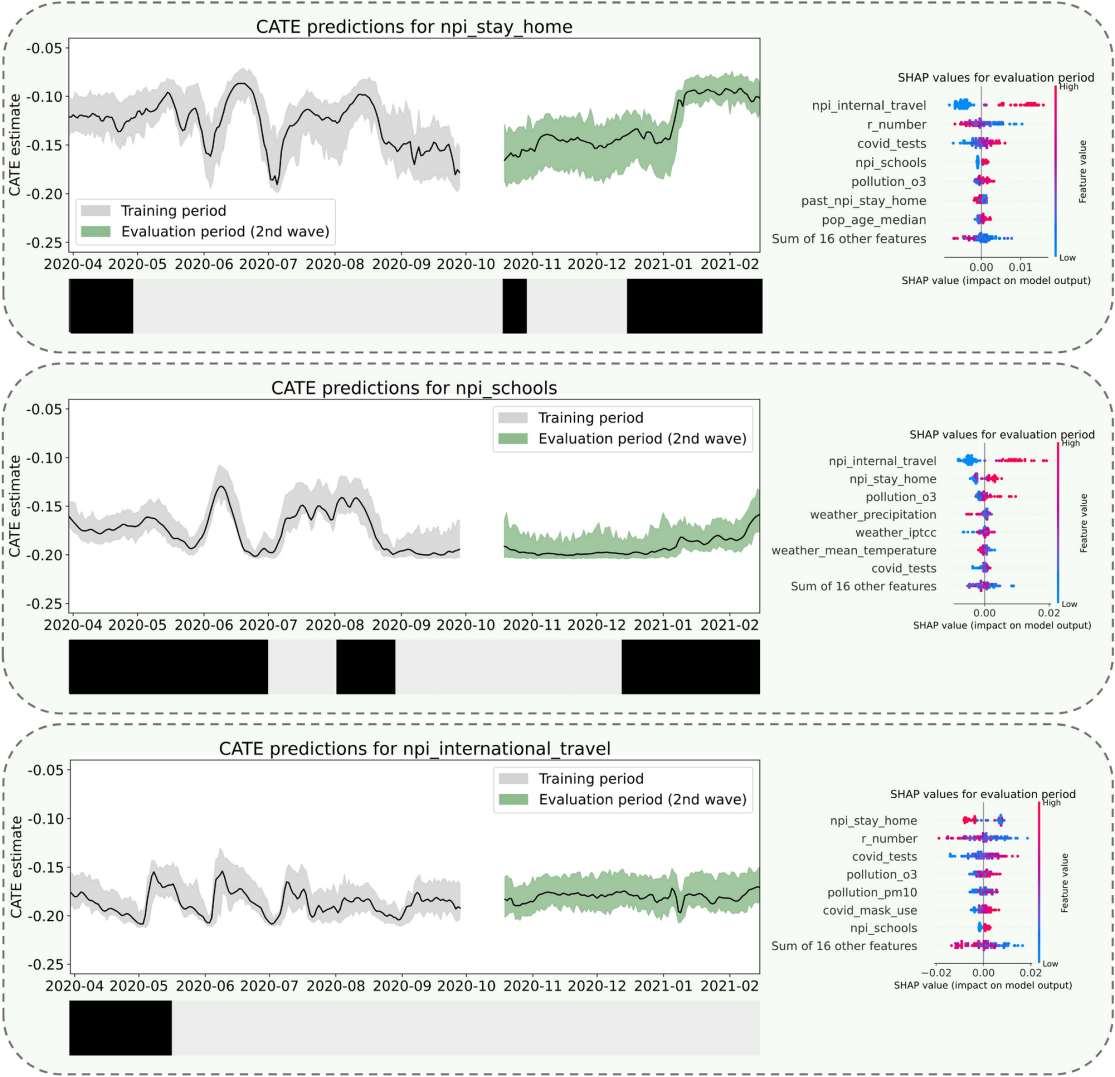
Scenario planning for the second wave in Germany. Models causal impact of stay-at-home orders (top panel), school closures (middle panel) and border closures (bottom panel) with the second wave held out for evaluation. The binary plots below CATE predictions depict the chronology of actually imposed NPIs. On the right, SHAP results are summarized for predictions in the evaluation period. SHAP values can be useful to gain a high-level intuition of feature importances, but are not guaranteed to reliably attribute the correct importance to every feature^45,46,48^.

More details on *absolute* CATE estimates and SHAP results for NUTS level 0 are given in Fig. 6, and predicted *relative* CATE on $R_t$ as well as current $R_t$ are plotted in Supplementary Fig. S6, for all NUTS level 0 and 1 units. Estimates are similar, but not identical across regions. Both stay-at-home orders and school closures are recommended as standalone measures with considerable effectiveness at the beginning of the wave. Stay-at-home policies are suggested rather specifically as an acute measure for high $R_t$ (reaching a minimium of −18 % relative CATE on $R_t$ in October 2020) with clearly dwindling effectiveness towards the end (leveling off between −12 and −13 %). School closures were modelled to effectively reduce lower current $R_t$ (arguably already curbed by previous NPIs) even further, with estimates arriving at a negative peak of relative CATE of −25 % in December 2020. At the end of the wave, there is also a decreasing trend in absolute and relative effectiveness. The predicted effect of border closures is comparably consistent over time (around −18 % relative CATE), but stronger while stay-at-home and internal travel regulations are also in place. Effects of internal travel restrictions as standalone interventions were predicted with equal consistency, but at a distinctly lower effect level around −4 %.

All models learned to focus on the other NPIs as the most influential predictors, which illustrates pronounced interdependence between the NPIs. Interestingly, internal movement restrictions were found to be the most important feature for both the npi_stay_home and the npi_schools model, even though the isolated causal effect for this policy seems comparably low. In addition, seasonal covariates like weather and air pollution were found to prominently affect CATE predictions in the second wave. For example, school closures seem to be more effective with higher temperatures, whereas higher air pollution reduces the effect.

## Discussion

We established a causal machine learning approach for estimating NPI effectiveness in observational public health data during a pandemic. In order to build a recommendation framework that helps decision makers in the planning of containment policies, it is important to account for the heterogeneity of NPI effects over time and between regions, and to address time-dependent confounding. If the latter is not considered, our refutation analysis disclosed severe overlap violations and effect estimates can be expected to be biased. This demonstrates the importance of monitoring model robustness such that mis-specifications are not overlooked. As a means to correct the bias arising from time-dependent confounding, we mapped input time series data to a treatment-invariant representation as suggested by Bica et al.^6^. Alternatives^15,16,18,50 ^and extensions^17^ of this approach may also be considered in future work; however, the objective of this work was the application of causal machine learning and not a detailed comparison of methods.

Due to comparably poor generalization of two-weeks ahead predictions in unseen evaluation data, we decided to base CATE estimates on a projection horizon of only seven days. Even though we do not expect NPIs to take full effect in this short time span, we argue that estimates should still be valid with our approach. NPIs are interventions that are adopted once and are then maintained for several weeks in a row. Thus, we can model and estimate the effect of an NPI being active over a certain period of time rather than the effect that the NPI has on $R_t$ in exactly one week.

A specific problem in the data is that NPIs are often imposed in bundles at a time. As a consequence, parts of the estimated effects that our models predicted for one NPI may in reality be partially attributable also to another. To a certain extent, including other NPIs as confounding and effect-modifying features in models with one specific NPI as treatment accounted for interaction between the different interventions in our modeling approach. The SHAP results in our scenario planning point to a high interdependence between the effectiveness of NPIs. We did not build one model for all treatments at the same time as was done in the original CRN publication because NPIs are very likely to be confounded at a present moment in time.

We also intentionally did not fit one model on the combined data from all countries, even though this would have given the models a larger training set. While Hale et al.^30^ encoded policy responses to a common scheme of categories across countries, we expect that the details of how NPIs were implemented still differ significantly internationally. Heterogeneity within a treatment may give rise to violations of the (untestable) assumption of consistency^51^, which may introduce a bias into the estimated effects.

From a historical perspective, our analyses corroborate that a wide range of response measures seems to have been effective up to a certain extent across different EU countries, including the trust-based policies in Sweden. However, not only the isolated effects on $R_t$ should play a role in the decision making process during acute pandemic situations, but also repercussions that a policy may have for social or economic variables. Furthermore, that an NPI is found to be effective in one country does not mean that it would be equally effective in another. For example, our results do not allow us to draw any conclusions as to whether the Swedish trust-based policy would have worked in the other countries, too. Even though recommendation-based policies were also in place in the other countries at times, they usually got tightened when new infection waves started to build up. As can be seen in column C of Supplementary Fig. S2, this entails that a lot of data has to be discarded for a recommendation analysis due to NPIs at above-recommendation levels. On an exploratory basis, we ran a full recommendation analysis for all countries with npi_stay_home_r and npi_internal_travel_r, where at least some data is available for a binarization across the seven countries and throughout the pandemic. Supplementary Fig. S7 demonstrates that effects can be found for some countries (Poland, the Netherlands, Belgium), but not for others (especially Germany). However, these results have to be considered with special caution because they rest upon very limited data, lacking information from crucial periods of the pandemic.

Compared to the models fitted on all available data from 2020 to 2022, models restricted to the first pandemic wave predicted distinctly larger effects for some NPIs. A possible explanation for this is that the initially high effectiveness of some NPIs subsided over time due to, e.g., waning public compliance with the policies, the availability of vaccines and increasing herd immunity.

Based on our study, we can make several recommendations considering data and modeling requirements for future efforts to predict the causal effect that NPIs may have, and thus advise policy makers how to invest in preparedness for new pandemics. Even though our models predicted that most NPIs lowered $R_t$ throughout the pandemic and across countries, surprisingly little heterogeneity over time was observed in the estimated effects. As a consequence, the predictions of models fitted on all available data from the whole pandemic period fail to represent the varying effectiveness over time that is evident from the models restricted to the first wave. In comparison to training on NUTS level 0 data alone, predicted heterogeneity tends to be larger when also taking level 1 data into account, which becomes particularly clear in Fig. 5 for the first wave. This could indicate that, to capture the time-dependent heterogeneity needed to inform dynamic response policies, it is beneficial to have subnational data available next to country-level data. A reason for this may be that the subnational units add more variability for all included features within periods with and without active NPI, compared to variability *between* these periods; in consequence, the outcome head of the CRN may put more relative importance on NPI status among all variables, and less variation of the other features has to be discarded in the balancing representation due to its distinctiveness of the current status of the NPI. This explanation is supported by the finding that there are positive correlations between the Shannon entropy of the NPI active vs. non-active status distributions over time and the 95 % boundaries of interval estimates over time: We observe Pearson correlation coefficients of 0.166, 0.315, and 0.110 for the boundaries plotted in Fig. 3, 4 and 5, respectively, and even 0.283, 0.383, and 0.263 for the left subplots based on NUTS level 0 data only. This reveals that lack of heterogeneity in the predictions is a problem that is not exclusively observed, but particularly pronounced for cases with an imbalanced NPI treatment variable, which might explain why no effect was found, e.g., for internal movement restrictions in the pseudo-prospective scenario planning analysis. Practically, all data should match the administrative hierarchy within a country at which NPI decisions are made, and ideally extend to more fine-grained regional resolution below that. However, it is equally important that high quality is ensured in both national and subnational data. We suspect that regional data in some countries (e.g., France and Spain) suffered from quality issues and thus decreased model robustness.

Moreover, it is advisable to consistently collect a wide range of possible confounding variables, which can be either included in causal models in the first place or later used in refutation tests for unobserved confounding.

Of course, our study is not without limitations. While we incorporated a broad array of potentially confounding and effect-modifying variables (other active NPIs, vaccination policy, COVID tests, mobility, air pollution, meteorological conditions, socio-economic conditions, demography) to estimate causal effects of individual NPIs, we cannot rule out that further factors may exist which we did not consider. This could, e.g., include policy fatigue and compliance with introduced NPIs. Even if a model passes our refutation testing, there can never be a guarantee that it would not produce qualitatively different results if the additional factors were included and that unobserved confounding is not ignorable after all. Estimating the robustness of causal models is generally a challenging task, which has not yet reached the same level of maturity as given for predictive machine learning models. Further research is needed in the community to reach a consensus about a standardized approach to evaluate causal machine learning models and report their performance.

A further limitation of our causal study is the quality of the data for both the treatment (NPIs) and outcome ($R_t$) variables.

In the case of the OxCGRT dataset of NPIs, the main issue is a lack of subnational resolution. In late 2020, many governments moved towards NPIs on a regional (NUTS 1 or 2) rather than a national basis^5^. However, the OxCGRT database always encodes the strictest level of an NPI anywhere within a state territory, regardless of the policies in other regions. In terms of causal identifiability, the consistency assumption may be violated within one country because treatments are qualitatively different across regions. To check the validity of our $R_t$-related CATE findings, we excluded all time points for which there was no way of knowing if an NPI was actually above the binarization threshold in the entire country (geo flag=1, if not directly following above-threshold time points with geo flag=0). Column B of Supplementary Fig. S2 shows which time points were discarded in this process, and which NPIs had to be dropped from the analysis in consequence. Supplementary Fig. S8 demonstrates that the results from the filtered data are qualitatively similar to those obtained based on the whole dataset, with most NPI effects being negative, but of moderate size. This validates our previous findings with a more conservative handling of available NPI information.

With regard to $R_t$, inaccuracies may arise due to either the quality of the underlying surveillance data or due to divergence in the estimation procedure. For example, asymptomatic cases are unlikely to be captured in any surveillance data, but they can substantially contribute to disease spread. The size of their portion in the total number of infections is difficult to estimate and may vary with different virus variants and seasonal influences. Moreover, data of confirmed cases is often noisy because of varying reporting rates that depend on changing testing capacities or policies. Especially in the early stages of the COVID pandemic, almost no tests were available, which very likely led to a severe underestimation of infection numbers. In our analyses, this becomes particularly clear in the comparison of predicted reduction of infection spread in the first wave, which is decidedly more pronounced for $R_t^{deaths}$ based on COVID-related death numbers (Fig. 5) than $R_t$ based on the number of confirmed cases (Supplementary Fig. S5). For France, Supplementary Fig. S5 even shows substantial *positive* effect estimates, which hints at poor quality of $R_t$ in the early phases of the pandemic. We accounted for this problem by including test rate as a time-dependent confounder, and by estimating $R_t^{deaths}$ as an alternative. While death data does not depend on the testing rate as confirmed cases data does, some other shortcomings have to be expected. On the one hand, the broad distribution of infection-to-death lags increases uncertainty as to whether a deceased patient contracted the disease during an NPI active or non-active period. Furthermore, the definition of who actually died “of” or “with” COVID may vary considerably between different regions (which may be a reason why our models learned to predict $R_t$ better than $R_t^{deaths}$ when subnational data was included). Finally, the number of cases and deaths in a population may be uncoupled to varying extents determined by, on the one hand, epidemiological factors such as virus variants and population seroprevalence, but also other factors like intensive care capacities or the success of response measures to specifically protect at-risk populations. For instance, our observations that $R_t$-based NPI effects in Germany partly vanished for $R_t^{deaths}$ may be explained by a successful strategy of the German government to protect and treat at-risk individuals. Although we used both $R_t$ and $R_t^{deaths}$ in our study as a mutual check of results validity, we have a preference for $R_t$ as a more common and direct statistic during an ongoing pandemic situation.

We validated our $R_t$ against independent estimates from different sources, namely Arroyo et al.’s^52^ results from an approach based on Kalman filters, plus public data published by the German, French and Dutch governments. Supplementary Table S4 shows that our estimates are generally correlated with Arroyo et al.’s results. Compared to Arroyo’s $R_t$, ours are a lot closer to those published for Germany and the Netherlands, but not for France. This shows that the choice of the $R_t$ estimation procedure may have a considerable impact on the outcome variable and, in turn, on the results of a causal analysis. For the sake of consistency across models, we decided to use the same algorithm for all regions and waves. However, we point out that, during an ongoing pandemic, the optimal solution may be specific to the present local situation.

## Conclusion

Based on historical data from the COVID-19 pandemic, we demonstrated how NPI effectiveness can be assessed in a retrospective and pseudo-prospective manner with the help of causal machine learning in seven European countries. On the basis of our results, we strongly recommend to account for time-dependent confounding and thoroughly check for violated assumptions with refutation analysis. We showed how our models could have been applied at a specific point in the pandemic in a prospective manner. According to our study, most NPIs are considered effective across countries, especially early on in the pandemic. When extending the same analysis to all available data from 2020 to 2022, effect sizes on $R_t$ appear only moderate.

To enhance the preparedness for future pandemic outbreaks, based on our analysis we recommend to routinely record public health data with a high quality, and at high temporal and spatial resolution. This would enable dynamic re-training and application of causal models to estimate NPI effects whenever necessary.

## Supplementary Information

Supplementary Information.

## Data Availability

The fully compiled and preprocessed dataset is provided - along with the code - at https://github.com/SCAI-BIO/causal-npi-effects. All raw data is publicly available. Case data was downloaded from the COVID-19 European regional tracker (https://github.com/asjadnaqvi/COVID19-European-Regional-Tracker). Death data had to be collected from national platforms individually: From the Robert Koch Institute for Germany (https://github.com/robert-koch-institut/COVID-19-Todesfaelle_in_Deutschland?tab=readme-ov-file), from data.gouv.fr for France (https://www.data.gouv.fr/fr/datasets/synthese-des-indicateurs-de-suivi-de-lepidemie-covid-19/), from the Centro Nacional de Epidemiología for Spain (https://cnecovid.isciii.es/covid19/#documentaci%C3%B3n-y-datos), gov.pl for Poland (https://www.gov.pl/web/koronawirus/wykaz-zarazen-koronawirusem-sars-cov-2), the National Institute for Public Health and the Environment for the Netherlands (https://data.rivm.nl/covid-19/), Sciensano for Belgium (https://epistat.sciensano.be/covid/), and Folkhälsomyndigheten for Sweden (http://fohm-app.folkhalsomyndigheten.se/Folkhalsodata/pxweb/sv/A_Folkhalsodata/A_Folkhalsodata__H_Sminet__covid19__falldata/ccov19Reg.px/). We used data on policy responses from the Oxford COVID-19 Government Response Tracker (https://github.com/OxCGRT/covid-policy-tracker), mask use estimates from the Institute for Health Metrics and Evaluation (https://www.healthdata.org/research-analysis/diseases-injuries/covid-research-library#download) and test rates from the European Center for Disease Prevention and Control (https://www.ecdc.europa.eu/en/publications-data/covid-19-testing). Google mobility data is available at https://www.google.com/covid19/mobility. Socio-economic and demographic data can be downloaded from Eurostat (https://ec.europa.eu/eurostat/data/database). We collected weather data from European Climate Assessment & Dataset (https://www.ecad.eu/dailydata/), the Royal Meteorological Institute of Belgium (https://opendata.meteo.be/downloadPage.php), and Météo France (https://donneespubliques.meteofrance.fr/?fond=produit&id_produit=90 &id_rubrique=32). Air pollution data is published by the European Environment Agency (https://discomap.eea.europa.eu/map/fme/AirQualityExport.htm).
