# Supplementary material for: Estimating the causal impact of non-pharmaceutical interventions on COVID-19 spread in seven EU countries via machine learning
Source: Sci Rep. 2025 Mar 17;15:9203. doi: 10.1038/s41598-025-88433-2 (PMC11914055; doi:10.1038/s41598-025-88433-2)
Supplement: Supplementary file 1 — Supplementary Information. [file 41598_2025_88433_MOESM1_ESM.pdf]

Supplementary material for ”Estimating the causal impact of non-pharmaceutical interventions on COVID-19 spread in seven EU countries via machine learning”

Jannis Guski[1*], Jonas Botz[1], Holger Fröhlich[1,2]

1 Fraunhofer Institute for Algorithms and Scientific Computing (SCAI), Department of Bioinformatics, Sankt Augustin, 53757, Germany
2 University of Bonn, Bonn-Aachen International Center for Information Technology (b-it), Bonn, 53115, Germany

* jannis.guski@scai.fraunhofer.de

Figure S1

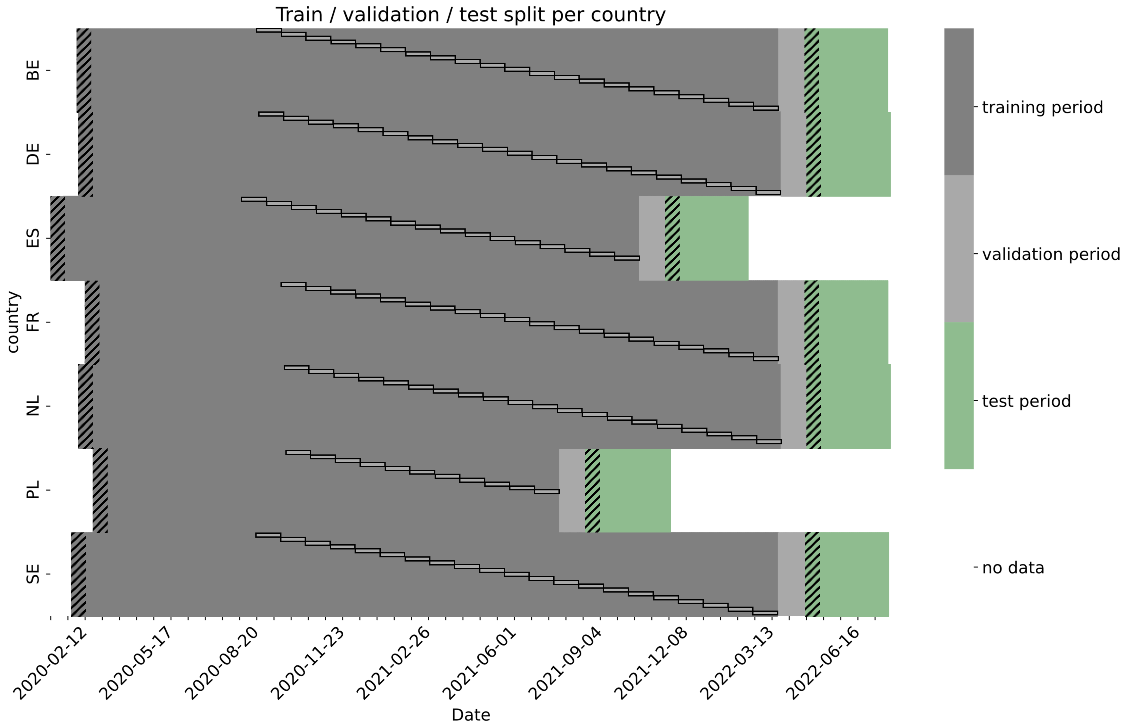

Overview of periods for which data was available per country. Hyperparameter optimization was performed within the training period with time-series cross validation (validation sets correspond to framed light gray boxes). Hatched areas were used in fitting, but not in prediction windows.

Figure S2

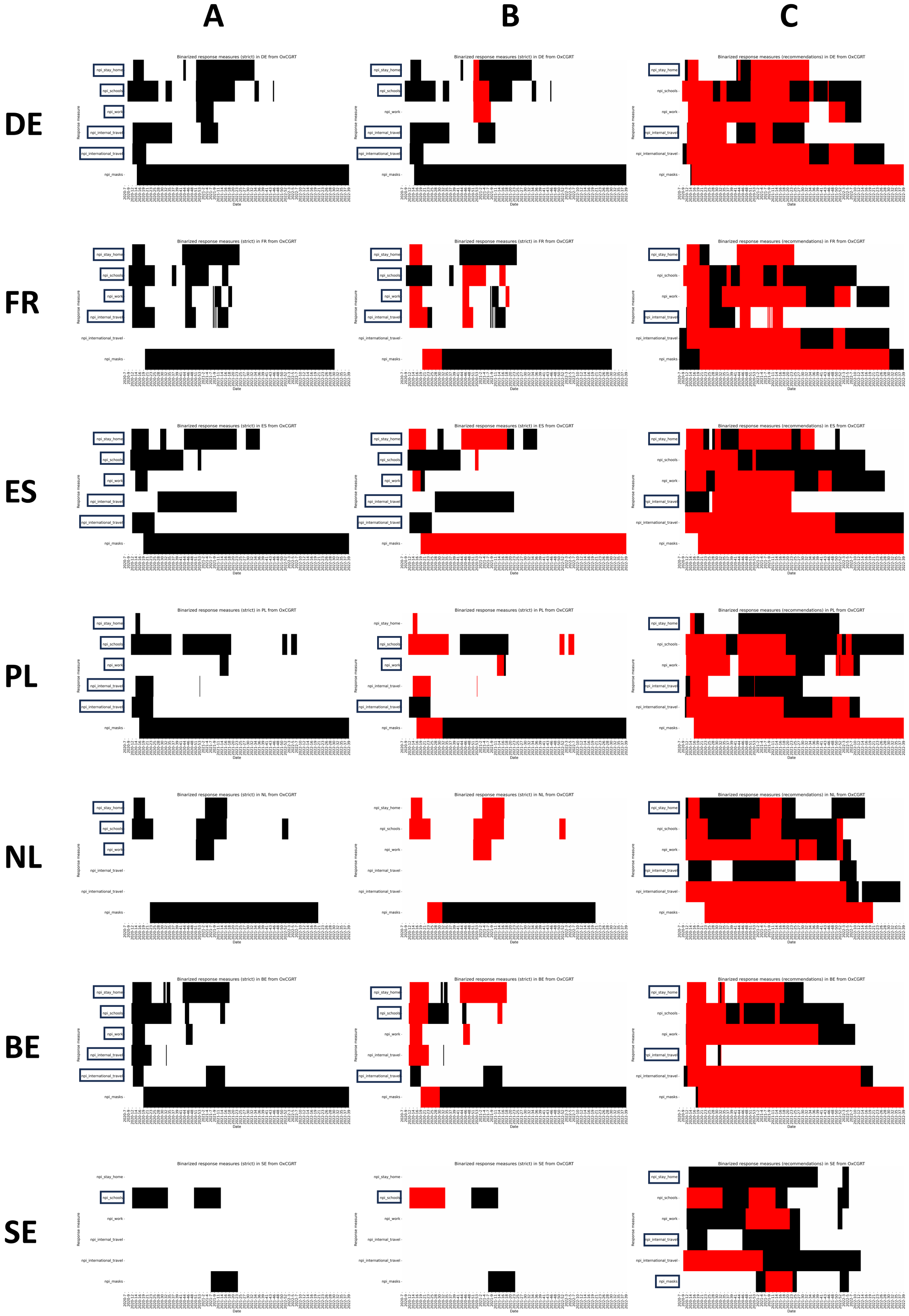

Overview of Non-Pharmaceutical Interventions (NPIs) from the Oxford COVID-19 Government Response Tracker (OxCGRT) used for different experiments. Chosen NPIs are bold-framed. Column A: "Required" measures binarization. Column B: "Required" measures binarization, filtered observation times by "geo flag" where it is uncertain if policy applied to whole country (marked in red). Column C: "Recommended" binarization, filtered observation times with

NPIs active at higher (”required”) level (marked in red).

Figure S3

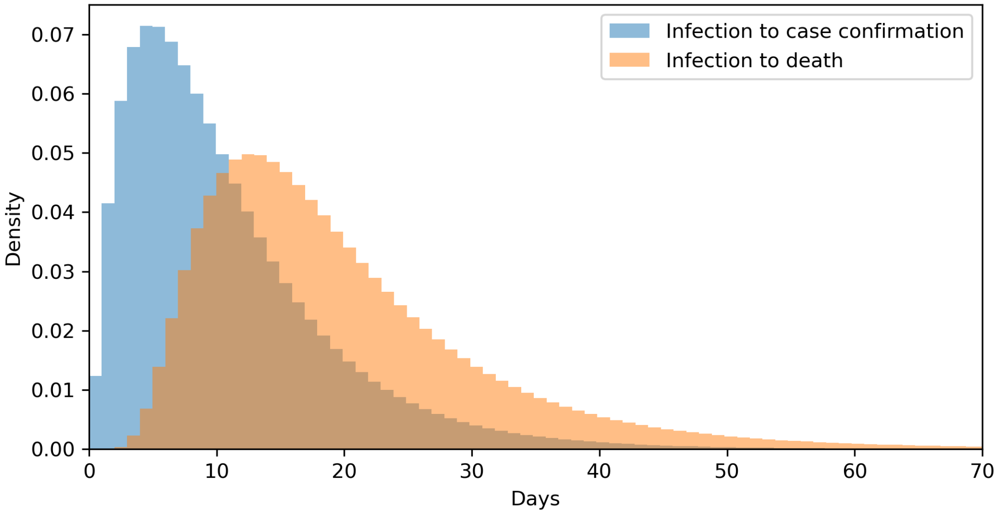

Delay distributions used for R_t and R_t^{deaths} estimation with `epyestim`. The distribution for infection to case confirmation (blue) is a convolution of a gamma distribution for incubation times and a negative binomial distribution for onset-reporting lag as reported by [1]. The distribution for infection to death (orange) is log-normal according to the modeling by [2] for the early phase of the pandemic in the United Kingdom.

Methods S1 Feature-specific preprocessing for the potentially confounding and effect-modifying variables The number of tests - only available at Nomenclature of Territorial Units for Statistics (NUTS) level 0 and weekly resolution - was scaled by the relative share of total cases attributable to a region to get NUTS 1 estimates, and weekly values were copied seven times to match the daily resolution of the outcome. Mask use projections were included at NUTS level 1 if available (only for Germany); otherwise, NUTS 0 data was used invariably for all regions. Likewise, NUTS 0 data on vaccination policy (available from the OxCGRT with predefined categories [3]) was utilized for all subnational units.

Google mobility trends estimate changes in mobility relative to a reference period before the pandemic, e.g., for residential, work, retail or transit areas. Due to substantial correlations between these, we included only transit areas which encompass public transport hubs like bus stops and train stations. As numbers of out-region and in-region commuters were found to be highly correlated, we only considered the former (for regions within the same and in a foreign country) and discarded the latter. Missing data on commuting to foreign countries in Bremen, Hamburg and Corsica was imputed with the mean across NUTS 1 regions of Germany and France, respectively.

Meteorological and air pollution data was available from surface weather stations spread over Europe. Based on their geographical coordinates, we mapped the stations into the NUTS units and mean-aggregated the reported features within each unit. Due to limited data availability or poor quality in the central European hub of stations, humidity data for France and all weather features for Belgium were collected separately. For some regions into which no stations could be mapped, data was copied from enclosing or adjacent regions: Both weather and pollution data for Madrid from the Centro region, pollution data for Brussels from the Flanders region, and humidity for the macroregion of Południowo-Zachodni from Południowy. Following [4], we also combined temperature and humidity to form the PREDICT index of climatic transmissibility of COVID-19 (IPTCC); this index is grounded on physical considerations and ranges from zero (low transmissibility weather

conditions) to one hundred (weather conditions facilitating the transmission of COVID-19). As shown in Table S1, there is a high positive correlation between our IPTCC variable over the whole time span of our input dataset and the sinusoidal multiplicative factor $\Gamma(t)$ that [5] used to model SARS-CoV-2 across seasons, even though we simply adopted the seasonal amplitude of the ”combined model” from that paper and optimized the day of the year with the highest seasonal effect based on correlation for the pooled dataset. This lends mutual validity to the IPTCC formula from [4], the sinusoidal modeling from [5] and the way that we collected weather data for each region. The correlations are notably smaller for Poland and especially Sweden, which may be due to less clear seasonal variation of temperature and humidity in these countries and a poor fit of γ or d_γ. However, the influence of seasonal weather conditions on COVID-19 spread is completely non-parametric in our approach and we expect our models to infer the correct relationships right from the data.

All features available only annually (number of commuters, gross domestic product per capita, hospital beds per 100,000, physicians per 100,000, share of households with broadband internet access, population density, population median age) were treated as static, using (if available) the value for 2020 or the most recent available value before that otherwise. Data on hospital beds and physicians, which was available only at NUTS level 2, was averaged within the coarser NUTS units of level 0 and 1. We discarded the feature of hospital beds for the Netherlands, to which we had no access at NUTS level 1.

The confounding or effect-modifying features were joined to R_t, and missing portions imputed firstly with cubic spline interpolation and secondly with forward / backward fills within regions.

	Correlation IPTCC and seasonal multiplier $\Gamma(t)$
pooled	0.656
DE	0.699
FR	0.846
ES	0.837
PL	0.512
NL	0.787
BE	0.755
SE	0.133

Table S1 Pearson correlation coefficients between IPTCC and the sinusoidal multiplicative factor $\Gamma(t)$ from [5] for the whole time span of our input dataset. Using the seasonal amplitude of $\gamma = 0.267$ from the combined model in [5] and day of the year with highest seasonal effect $d_\gamma = 32$, which optimizes the correlation for the pooled dataset.

Hyperparameter	Search space	Final choice
Batch size	[32, 64, 128, 256]	64
Learning rate	(1e-5, 1e-3)	5.97e-4
Dropout (Input)	(0, 0.9)	0.042
Dropout (LSTM)	(0, 0.6)	0.234
L1 lambda	(1e-5, 1e-2)	1.12e-5
L2 lambda	(1e-5, 1e-2)	1.18e-4
Latent representation size	[16, 32, 64, 128]	64
Number of layers (LSTM)	[1, 2]	2
Hidden layer size (LSTM)	[16, 32, 64, 128]	128
Hidden layer size (FC)	[16, 32, 64, 128]	128
Window size	[7, 14, 21]	14

Table S2 Optimized hyperparameters used for CRN fitting in pseudo-prospective scenario planning for second wave in Germany. LSTM, Long Short-Term Memory; FC, Fully Connected

Identified with tree-structured Parzen estimator from `Optuna` and time-series cross validation.

Figure S4

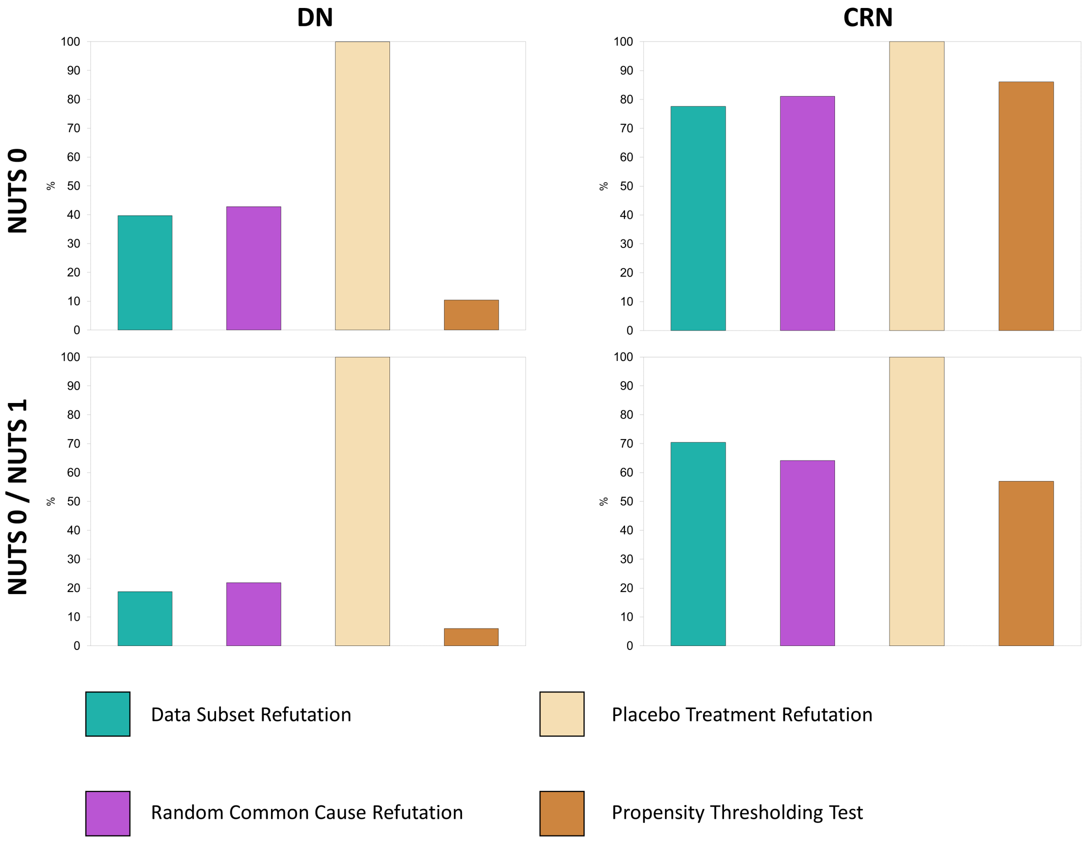

Model robustness in training data according to refutation analysis. Portions of observations that passed each refutation test in data used for training, comparing Dragonnet (DN) (left) vs. Counterfactual Recurrent Network (CRN) (right) and NUTS level 0 only (top) vs. levels 0 and 1 combined (bottom).

	7-days ahead		14-days ahead	
	Train	Eval	Train	Eval
R_t				
NUTS 0	8.47 % (±0.02 %)	15.99 % (±0.06 %)	9.26 % (±0.02 %)	17.69 % (±0.07 %)
NUTS 0 + 1	10.64 % (±0.02 %)	13.69 % (±0.11 %)	10.77 % (±0.03 %)	22.92 % (±0.14 %)
R_t^{deaths}				
NUTS 0	7.87 % (±0.01 %)	14.28 % (±0.05 %)	8.25 % (±0.02 %)	18.38 % (±0.07 %)
NUTS 0 + 1	15.03 % (±0.03 %)	17.71 % (±0.09 %)	15.76 % (±0.03 %)	20.56 % (±0.13 %)

Table S3 Mean Average Percentage Error (MAPE) for factual R_t in training and evaluation data. Aggregated over NPIs and countries. We distinguish between models that estimate Conditional Average Treatment Effect (CATE) based on 7-days and 14-days ahead

predictions, and models fitted on NUTS 0 units only and on both NUTS 0 and NUTS 1. For a fair comparison, MAPE is computed only for NUTS 0 data.

MAPE is defined as [6]

$$MAPE = \frac{1}{n} \sum_{i=1}^{n} \left| \frac{A_i - F_i}{A_i} \right| \times 100$$

Where:
- n is the number of data points
- A_i is the actual value
- F_i is the forecasted value

Figure S5

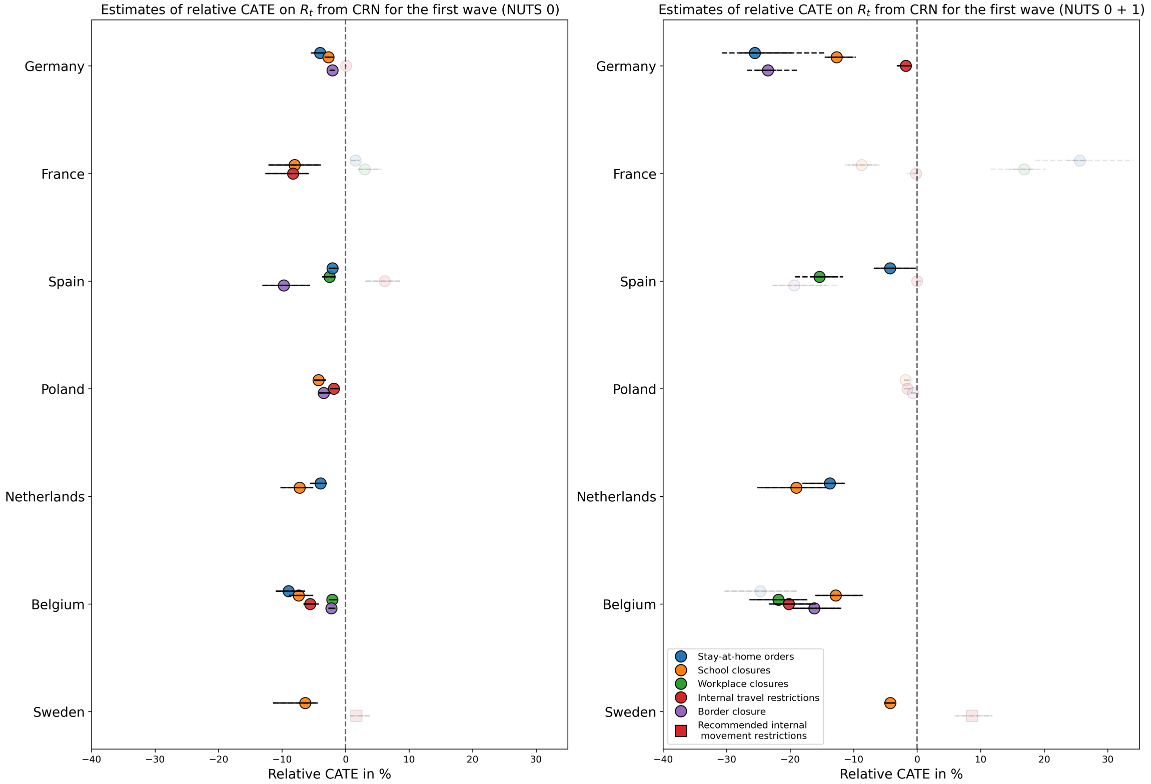

Distributions of predicted relative CATE on R_t in the first wave from CRN over time per NPI and per country. Left: NUTS level 0 only, right: Fitted with levels 0 and 1 combined, but predictions only for NUTS 0. Solid lines denote the 95 % CI of point estimates over time, dashed lines the 95 % CI of lower and upper boundaries of interval estimates over time. Markers are greyed out if CATE is non-significant or any refutation test is failed by 50 % or more observations.

Figure S6

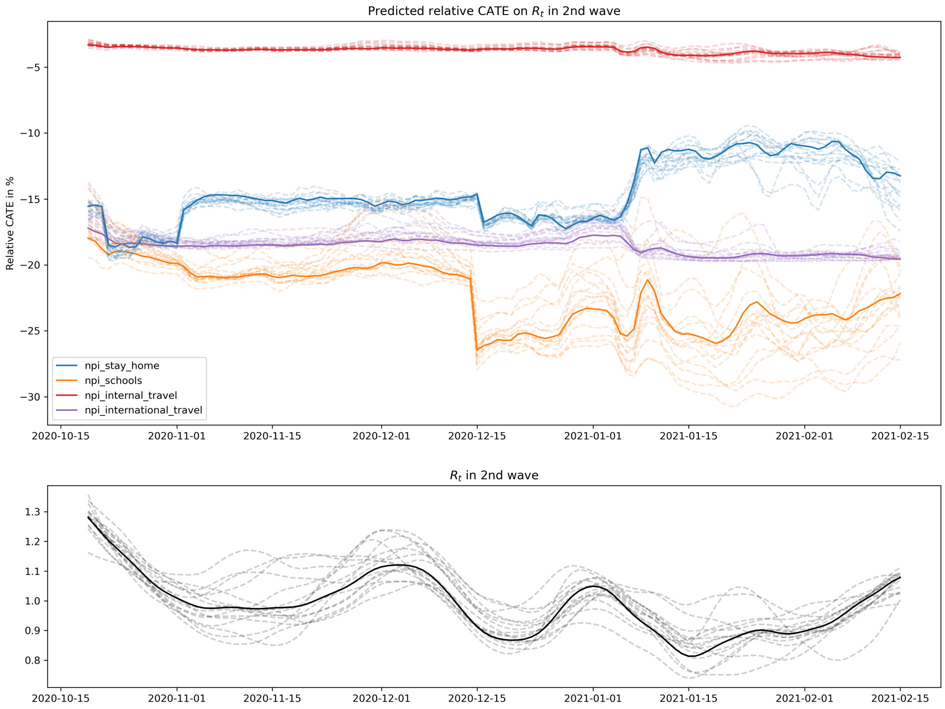

Relative predicted CATE on R_t in the second wave in Germany as modeled in the retrospective scenario planning analysis. Bold lines are for NUTS level 0, dashed lines for NUTS level 1 (Bundesländer). Upper subplot: Relative reduction in R_t. Lower subplot: R_t.

Figure S7

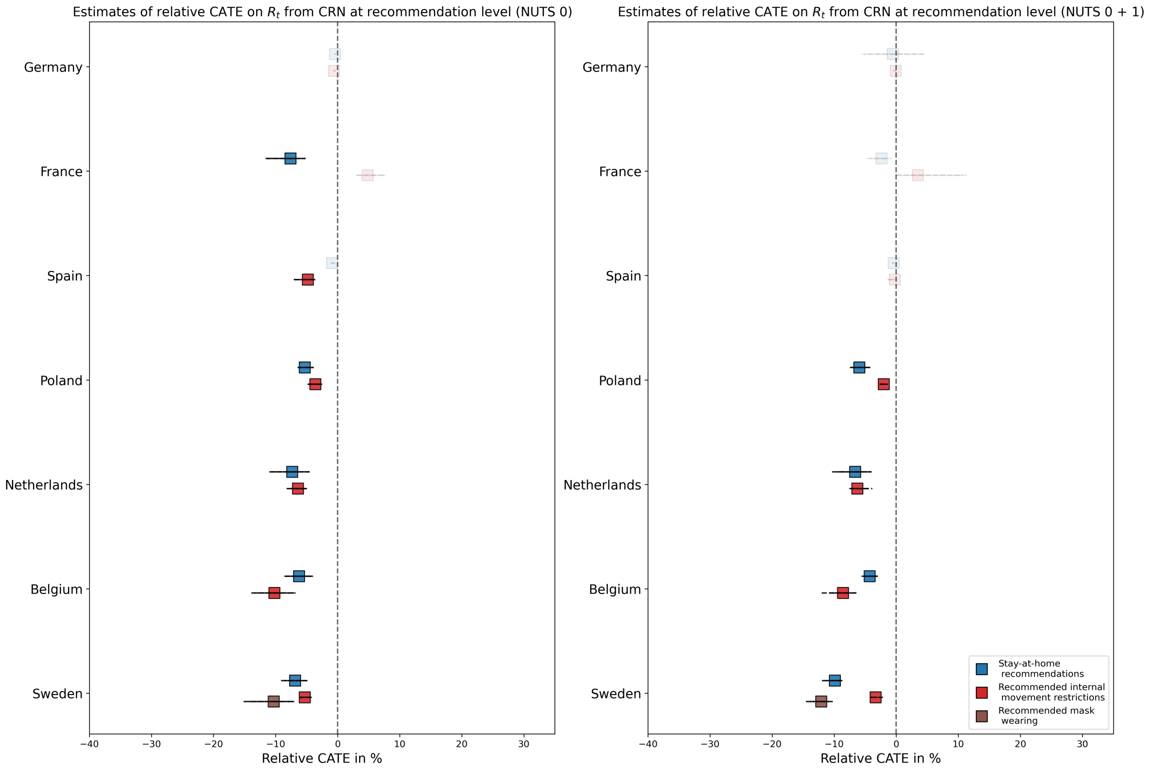

Distributions of predicted relative CATE on R_t from CRN over time per NPI and per country for recommendation analysis. Left: NUTS level 0 only, right: Fitted with levels 0 and 1 combined, but predictions only for NUTS 0. Solid lines denote the 95 % CI of point estimates over time, dashed lines the 95 % CI of lower and upper boundaries of interval estimates over time. Markers are greyed out if CATE is non-significant or any refutation test is failed by 50 % or more observations.

Figure S8

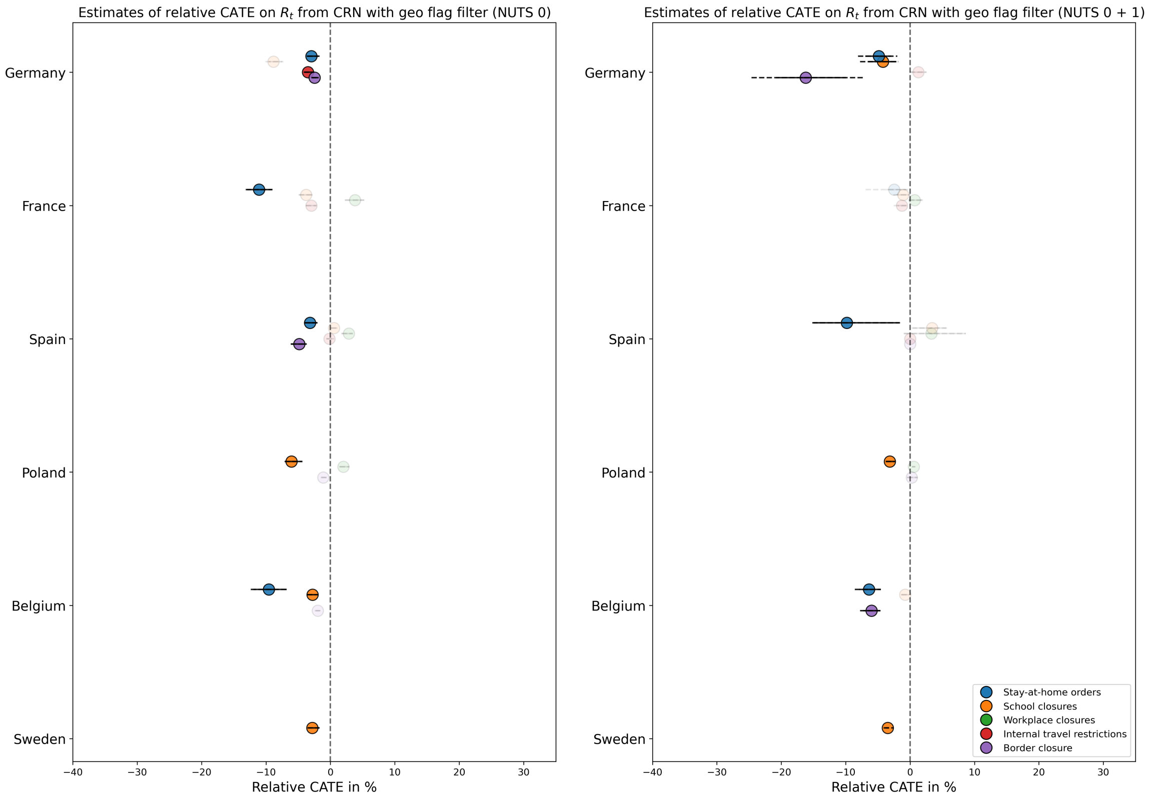

Distributions of predicted relative CATE on R_t from CRN over time per NPI and per country, filtered based on OxCGRT geo flag. Left: NUTS level 0 only, right: Fitted with levels 0 and 1 combined, but predictions only for NUTS 0. Solid lines denote the 95 % CI of point estimates over time, dashed lines the 95 % CI of lower and upper boundaries of interval estimates over time. Markers are greyed out if CATE is non-significant or any refutation test is failed by 50 % or more observations.

Correlations	DE_epyestim*	DE_arroyo**	FR_epyestim*	FR_arroyo**	ES_epyestim*	NL_epyestim*	NL_arroyo**	BE_epyestim*	SE_epyestim*	PL_epyestim*
DE_arroyo**	0.54									
DE_public***	0.84	0.78								
FR_arroyo**			0.43							
FR_public****			0.50	0.96						
ES_arroyo**					0.40					
NL_arroyo**						0.49				
NL_public*****						0.80	0.48			
BE_arroyo**								0.61		
SE_arroyo**									0.36	
PL_arroyo**										0.71

Table S4 Spearman correlations of our R_t estimates (epyestim) with independent estimates from different sources.

* Estimate used in our paper, based on epyestim package [7] and the COVID-19 European Regional Tracker [8]

** Reference estimates from [9] with 7-days serial intervals

*** Public estimates from the Robert Koch Institute (`https://github.com/crondonm/TrackingR`)

**** Public estimates from `data.gouv.fr` (`https://www.data.gouv.fr/fr/datasets/synthese-des-indicateurs-de-suivi-de-lepidemie-covid-19/`)

***** Public estimates from the Dutch National Institute for Public Health and the Environment (`https://data.rivm.nl/meta/srv/dut/catalog.search#/metadata/ed0699d1-c9d5-4436-8517-27eb993eab6e`)

References

1. Brauner JM, Mindermann S, Sharma M, Johnston D, Salvatier J, Gavenčiak T, et al. Inferring the effectiveness of government interventions against COVID-19. Science. 2021;371(6531). doi:10.1126/science.abd9338.

2. Ward T, Johnsen A. Understanding an evolving pandemic: An analysis of the clinical time delay distributions of COVID-19 in the United Kingdom. PLOS ONE. 2021;16(10 October 2021):1–15. doi:10.1371/journal.pone.0257978.

3. Hale T, Angrist N, Goldszmidt R, Kira B, Petherick A, Phillips T, et al. A global panel database of pandemic policies (Oxford COVID-19 Government Response Tracker). Nat Hum Behav. 2021;5(4):529–538. doi:10.1038/s41562-021-01079-8.

4. Roumagnac A, de Carvalho Filho E, Bertrand R, Banchereau AK, Lahache G. Étude de l'influence potentielle de l'humidité et de la température dans la propagation de la pandémie COVID-19. Médecine de Catastrophe - Urgences Collectives. 2021;5(1):87–102. doi:10.1016/j.pxur.2021.01.002.

5. Gavenčiak T, Monrad JT, Leech G, Sharma M, Mindermann S, Bhatt S, et al. Seasonal variation in SARS-CoV-2 transmission in temperate climates: A Bayesian modelling study in 143 European regions. PLoS Comput Biol. 2022;18(8):1–14. doi:10.1371/journal.pcbi.1010435.

6. Roberts A. Mean Absolute Percentage Error (MAPE): What You Need To Know;. Available from: `https://arize.com/blog-course/mean-absolute-percentage-error-mape-what-you-need-to-know/`.

7. Hilfiker L, Josi J. epyestim; 2021. Available from: `https://pypi.org/project/epyestim/`.

8. Naqvi A. COVID-19 European regional tracker. Sci Data. 2021;8(1):181. doi:10.1038/s41597-021-00950-7.

9. Arroyo-Marioli F, Bullano F, Kucinskas S, Rondón-Moreno C. Tracking R of COVID-19: A new real-time estimation using the Kalman filter. PLOS ONE. 2021;16(1 January):1–16. doi:10.1371/journal.pone.0244474.
